# Quality of clinical practice guidelines and recommendations for the management of pain, sedation, delirium and iatrogenic withdrawal in pediatric intensive care: a systematic review protocol

**DOI:** 10.1136/bmjpo-2021-001293

**Published:** 2022-02-15

**Authors:** Ibo MacDonald, Marie-Hélène Perez, Vivianne Amiet, Alexia Trombert, Anne-Sylvie Ramelet

**Affiliations:** 1 University Institute of Higher Education and Research in Healthcare, University of Lausanne Faculty of Biology and Medicine, Lausanne, Switzerland; 2 Department of Woman Mother and Child, Lausanne University Hospital, Lausanne, Switzerland; 3 Medical Library, Lausanne University Hospital, Lausanne, Switzerland

**Keywords:** pain

## Abstract

**Introduction:**

Inadequate management of pain and sedation in critically ill children can cause unnecessary suffering and agitation, but also delirium and iatrogenic withdrawal. It is, therefore, important to address these four interrelated conditions together. Some clinical practice guidelines (CPGs) are available for the management of pain and sedation, and a few for delirium and iatrogenic withdrawal in the paediatric intensive care unit; none address the four conditions altogether. Critical appraisal of the quality of CPGs is necessary for their recommendations to be adopted into clinical practice. The aim of this systematic review is to identify and appraise the quality of CPGs and recommendations for management of either pain, sedation, delirium and iatrogenic withdrawal.

**Methods and analysis:**

Researchers will conduct a systematic review in electronic databases (Medline ALL (Ovid), Embase.com, CINAHL with Full Text (EBSCO), JBI EBP Database (Ovid)), guideline repositories and websites of professional societies to identify CPGs published from 2010 to date. They will then combine index and free terms describing CPGs with pain, sedation, delirium and withdrawal. The researchers will include CPGs if they can be applied in the paediatric intensive care population (newborns to 18 years old) and include recommendation(s) for assessment of at least one of the four conditions. Two independent reviewers will screen for eligibility, complete data extraction and quality assessments using the Appraisal of Guidelines for Research and Evaluation (AGREE) II and the AGREE Recommendation Excellence instruments. Researchers will report characteristics, content and recommendations from CPGs in tabulated forms.

**Ethics and dissemination:**

Ethical approval is not required for this systematic review. Results will be published in a peer-reviewed journal.

**PROSPERO registration number:**

CRD42021274364.

**What is already known on this topic**

- Optimal analgesia and sedation management is challenging in the paediatric intensive care unit.
- Pain, sedation, delirium and iatrogenic withdrawal are interrelated conditions that need to be assessed as a whole to prevent negative outcomes.

**What this study hopes to add**

- Researchers will compare the quality of clinical practice guidelines across all four interrelated conditions of pain, sedation, delirium and iatrogenic withdrawal.
- Researchers will compare recommendations by describing the certainty and applicability of the base of evidence for clinical practice guidelines.

## Introduction

Critically ill children in the paediatric intensive care unit (PICU), particularly those who are mechanically ventilated, require adequate identification and treatment of pain, sedation, delirium and iatrogenic withdrawal.^1^ Healthcare professionals (HCPs) manage this with the use of analgesics and sedatives, often in combination, with 65% of children receiving both.^2^ Optimal titration of analgesics and sedatives prevent pain, delirium and iatrogenic withdrawal,^3 4^ as well as agitation that can cause accidental extubation.^5^ Over sedation and analgesia can result in prolonged mechanical ventilation, increased PICU length of stay, morbidity and mortality.^1^ In the PICU setting, 40%–65% of children are unable to self-report due to mechanical ventilation and young age (under the age of 4).^6 7^ Given the large proportion of non-communicative children and the need for quality care, it is crucial that HCPs use multiple measurement instruments to assess pain, sedation, delirium and iatrogenic withdrawal.

Assessment is the cornerstone for management of the four conditions.^8^ It helps HCPs to individualise treatment and plan appropriate multimodal interventions.^1^ Although, HCPs have access to several measurement instruments for assessing pain and sedation,^9^ and more recently, for delirium and iatrogenic withdrawal.^10^ A recent survey including 168 PICUs in 18 countries, found wide variation in the application of measurement instruments across the four conditions into practice.^11^ In fact, some researchers have shown that HCPs struggle to select the right measurement instrument for these four conditions.^12 13^ This may be due to the overlap among similar behavioural cue items across measurement instruments across these four conditions, or the plethora of measurement instruments available.^10 14 15^ While on one hand there is a need for psychometrically sound measurement instruments for each condition, it is equally important not to look at these conditions in siloes but to incorporate them together into standardised care practices,^14 15^ either in clinical practice guidelines (CPGs) or as protocols or algorithms to improve assessment and management.

CPGs are created by synthesising research to help bridge the evidence-to-practice gap. The purported benefits of CPG implementation are the standardisation of care practices; improvements in patient safety; and patient outcomes.^16^ There are few CPGs for management of these four conditions in the PICU, with only two for pain,^17 18^ and one for pain, sedation and delirium.^19^ Furthermore, some guidance documents exist, including practice/position/consensus statements/recommendations (hereafter, referred to as CPGs).^14 20 21^ Although there is one position statement for the assessment of pain, sedation, delirium and iatrogenic withdrawal for paediatric critical care patients,^14^ it does not include recommendations for management. Previous systematic reviews of CPGs for pain in paediatrics exist. They target neonates,^22^ or burn patients,^23^ or focus on procedural^22 24^ or acute pain.^23^ Systematic reviews of CPGs concerning best practices for children in the PICU are lacking. To date, none have been conducted on either of the four conditions, nor have they been examined together. Recently, scholars have criticised systematic reviews of CPGs and their lack of quality appraisal of recommendations.^25^ Several systematic reviews of CPG recommendations have demonstrated evidence that weakly supports the recommendations.^26 27^ Researchers, in a recent systematic review of CPGs for paediatric populations, that filtered publications between 2017 and 2019, found that 75% of the 216 CPGs were evidence-based.^28^ It is important that researchers establish methodological quality of CPGs and the evidence base of recommendations to promote evidence-informed interventions.

Quality CPGs and their contained recommendations should reflect the most current evidence. Although, numerous appraisal instruments exist for assessing the quality of CPGs,^29^ internationally, the appraisal of guidelines for research and evaluation (AGREE) II has emerged as the most widely used appraisal instrument.^30^ The AGREE Enterprise recently developed the AGREE recommendation of excellence (AGREE-REX) instrument for the quality appraisal of recommendations.^31^ Thus, researchers should use these two instruments jointly to assess CPG quality and to ensure that the evidence supporting recommendations in CPGs is reliable and trustworthy. This is essential so that CPGs remain sources of information that clinicians use to improve their practice and care of patients. The identification, appraisal and comparison of quality of CPGs and their recommendations is a valuable first step in informing efforts to incorporate these four overlapping conditions together in a standardised way to optimise care in the PICU. In this systematic review, researchers aim to appraise the quality of CPGs and recommendations for the assessment and management of pain, sedation, delirium and iatrogenic withdrawal in the PICU. Their specific objectives are:

1. To identify published CPGs for the assessment and management of pain, sedation, delirium and iatrogenic withdrawal.
2. To appraise the quality of selected CPGs.
3. To appraise the quality of recommendations included in CPGs.
4. To summarise the convergence of recommendations and the overall robustness of recommendation in CPGs.

## Methods and analysis

Researchers of this study protocol used the methodological guide for conducting systematic reviews of CPGs to guide the development of each stage.^32^ They reported according to the Preferred Reporting items for Systematic Reviews and Meta-Analyses protocols (PRISMA) (online supplemental table 1A).^33^

10.1136/bmjpo-2021-001293.supp1 Supplementary data

### Inclusion/exclusion criteria for study selection

To guide CPG selection, the population, intervention, comparators, attributes and recommendations framework was used (see table 1).^32^ For the purpose of this review, CPGs must have included recommendations developed from available evidence, including expert opinion.^16^ CPGs and guidance statements will be considered. CPGs that include paediatric populations will be included in this review if they: (1) are endorsed by a society, (2) include a recommendation for assessment of any of the four conditions and (3) are the most current version. Publication year will be limited to 2010 to present for two reasons. First, this timeframe corresponds with a paradigmatic shift in intensive care unit sedation practice.^34^ Second, the first consensus guideline for critically ill children was published in 2006.^20^ If updated within 5 years, as recommended,^35^ it would be captured within the search strategy. This review will include broad CPGs for the assessment and management of any of the four conditions, including postoperative pain. However, CPGs will be excluded if focused on specific patient groups (eg, cardiac). CPGs on diagnostic procedures (eg, endoscopy) or procedures of limited temporal duration (eg, venipuncture) will be excluded. Procedures that require prolonged use in the PICU setting (eg, respiratory support), will be included.

**Table 1:** PICAR statement: inclusion and exclusion criteria

Population, clinical indication(s), and condition(s)	**Study population**: Include: Children (newborn (>38 weeks gestations) to 18 years of age) Exclude: premature infants and adults **Clinical indications**: Include: management of either pain (including postoperative, persistent and prolonged pain), sedation, delirium or iatrogenic withdrawal Exclude: Management specific to medications, chronic and procedural pain of short duration, procedural sedation provided in other care settings (eg, dentistry, radiology, endoscopy) or for short duration **Conditions**: Include: children in intensive care
Interventions	Any intervention focusing on the on-going management of either pain, sedation, delirium or iatrogenic withdrawal
Comparator(s), comparison(s) and (key) content	**Comparator/comparison:** Any **Key content**: If a broad population is included, the CPG must have separate recommendations for children The CPG can be implemented in the intensive care setting but does not need to be specifically developed for intensive care The CPG must include recommendations on assessment for either pain, sedation, delirium or iatrogenic withdrawal
Attributes of eligible CPGs	**Language:** No restrictions **Year of publication**: 2010 onward **Setting**: Include: Applicable to paediatric intensive care, can be broad/general Exclude: CPGs developed specifically for other settings: neonatal intensive care units, emergency department, pre-operative/operating room **Developing/publishing organisation:** Include: CPGs issued or endorsed by international, national or regional societies/professional organisations, or governments from developed countries Exclude: CPGs that were developed by an individual organisation (eg, hospital) or unit within an organisation **Version**: Latest/newest version (preceding versions will be excluded) **Type**: CPGs, consensus statements, practice/position recommendations/alerts/statements **Quality score**: The AGREE II will be used to assess quality but will not be used as a criterion to determine eligibility for inclusion in this systematic review
Recommendations characteristics and ‘other’ considerations	**Recommendations:** CPGs must have at least one specific recommendation for assessment for either pain, sedation, delirium or iatrogenic withdrawal (either explicitly highlighted as a recommendation (primary) or noted in the body of the text (secondary—not explicitly identified as a recommendation))

AGREE, Appraisal of Guidelines for Research and Evaluation; CPG, clinical practice guideline; PICAR, population, intervention, comparators, attributes and recommendation.

### Search methods

#### Informational sources

The search will be conducted in:

1. Four electronic databases: Medline ALL (Ovid), Embase.com CINAHL with Full Text (EBSCO), and Joanna Briggs Institute (JBI) EBP Database.
2. Ten guideline repositories.
3. Thirteen professional societies/organisations (online supplemental table 1B) contains a list of guideline repositories and professional societies/organisations to be searched).

#### Search strategy

The search strategy will be developed with the assistance of a health services librarian. Index and free terms describing CPGs and pain, sedation, delirium, withdrawal will be combined to create an advanced search strategy that will be translated for all databases and sources of information. The final search strategy for Embase.com is provided in online supplemental table 1C. The search strategy will be peer reviewed by another librarian using the PRESS checklist.^36^ During full-text screening, if a CPG is mentioned, it will be retrieved for review.

#### Guideline selection

The search results will be imported into Endnote 20 reference manager (Clarivate Analytics, USA) for duplicate removal. The remaining citations will be uploaded to Rayyan QCRI (Qatar Computing Research Institute, Doha, Qatar) to manage the screening process.^37^

Titles and abstracts of all citations will be screened by two independent reviewers to determine those for full-text review. These will be retrieved and assessed against the inclusion and exclusion criteria. Reasons for exclusion will be recorded. Any disagreements will be resolved through discussion and consensus or by a third reviewer.

Supporting documents (eg, evidence tables, conflict of interest declarations), where available, will be retrieved by the review team from the endorsing organisation’s website to ensure all relevant documents will be available for quality appraisal for included CPGs.

The PRISMA flow diagram will be used to show the selection process and summarise the inclusion and exclusion details.^38^

### Data extraction

Information from each included CPGs will be extracted by two independent reviewers. The review team developed an Excel spreadsheet for data extraction (online supplemental figures 1–4D) to 4D) that will be piloted and revised during the data extraction phase. The following key areas will be extracted: (1) General information: title, first author, year of publication, language, developing organisation, country, type of CPG, condition addressed (pain, sedation, delirium, iatrogenic withdrawal), target population, target setting, level of evidence (LoE) rating system, grade of evidence rating system; (2) Quality of included CPGs using the AGREE II instrument (details below)^30^; (3) Quality of recommendations from medium quality and higher CPGs using the AGREE-REX instrument (details below)^31^ and (4) Recommendations (one worksheet per condition, and each line will represent one recommendation): recommendation, grade of recommendation, classification of evidence, list of supporting citation(s), categorisation of recommendation as per CPG.

### Quality appraisal of CPGs and recommendations

Quality of CPGs. Each included CPG will be independently appraised by at least two reviewers using the AGREE II instrument.^30^ The AGREE II is a validated and reliable appraisal instrument for assessing the quality of CPGs.^30^ It contains 23 items across 6 domains: (1) scope and purpose; (2) stakeholder involvement; (3) rigour of development; (4) clarity of presentation; (5) applicability and (6) editorial independence. Each item will be appraised against a 7-point Likert scale ranging from 1 (strongly disagree) to 7 (strongly agree). In addition, there are two global rating scores: (1) overall quality of the CPG, and (2) whether the guideline would be recommended for use.

Quality of CPGs recommendations. The AGREE–REX will be used for assessing the quality of recommendations.^31^ It is a recently developed, valid and reliable appraisal instrument containing nine items across three domains: (1) clinical applicability; (2) values and preferences and (3) implementability.^31^ Each item will be appraised by at least three reviewers using the same seven-point Likert scale, as in the AGREE II. It includes a global rating score of the overall quality of the CPGs recommendations.

To score the AGREE II each item’s score across reviewers will be summed and converted to a percentage of the maximum possible score for each domain.^30^ For the AGREE-REX, the consensus score approach will be used, whereby the review team will meet to agree on AGREE-REX item scores^39^

To ensure standardisation of appraisal, the training tools available for the AGREE II on the website (www.agreetrust.com) will be used to train each reviewer. For the AGREE-REX, a training video will be created by one research team member (ID). This video will be used to train each member of the review team. One included CPG will then be selected by the entire review team, and a consensus meeting will be held to ensure familiarity with the tools. The AGREE II inter-rater agreement will be calculated using intraclass correlation coefficients (ICCs) with a two-way random effects model for each domain. The levels of ICC agreement will be classified as poor (<0.50), moderate (0.50–0.75), good (0.75–0.9) and excellent (>0.9).^40^

### Data synthesis

#### Quality of CPGs and recommendations

The AGREE Enterprise has no established quality threshold, instead review teams must establish their own prior to appraisal.^41^ As recommended in a recent systematic review of AGREE II thresholds for determining CPG quality, we will use the three-step system where high quality are scores>60%, medium quality are scores between >30% and 60%, and low quality are scores <30% across all domains.^42^ For determining when the AGREE-REX will be applied to assessing recommendations the same a priori establishment of a threshold is recommended in the AGREE-REX user manual.^41^ For this review, the AGREE-REX will be used only with CPGs that meet at least the medium level threshold (eg, *>*30%) for methodological development using the AGREE II. Clusters of recommendations on single topics (eg, assessment) will then be appraised in these CPGs. This decision was made because this is the first review of its kind, and the quality of recommendations between CPGs is uncertain.

The results of the AGREE II and AGREE-REX scores will be presented in a table. The quality of each domain will be presented as a heat map based on the threshold cut-offs for quality as described above.

#### Synthesis of recommendations and their LoE

For medium and high methodological quality CPGs (based on the AGREE II), all recommendations and their related evidence will be extracted per condition. Once extracted, each recommendation will be categorised based on the type of care intervention, including: (1) prevention, (2) assessment and (3) management. Management interventions will be further subdivided into pharmacological and non-pharmacological. Summary tables will be created to highlight the consistency of all recommendations for each condition. The LoE associated with recommendations within each CPG will be reported but not standardised across CPGs.

## Patient and public involvement

There was no patient or public involvement in the development of the systematic review protocol. The Swiss Society of Intensive Care Medicines’ Pain, Agitation, Delirium, Immobility and Sleep working group will be involved in data synthesis as clinical experts.

## Discussion

This systematic review of CPGs will generate a succinct and comprehensive summary of the best available evidence for the assessment and management of pain, sedation, delirium and iatrogenic withdrawal. This will be a valuable first step towards standardising the assessment and management of pain, sedation, delirium and iatrogenic withdrawal in the PICU.

Research on pain, sedation, delirium and iatrogenic withdrawal practices across an international sample of 161 PICUs continues to demonstrate great variation.^11^ Although HCPs use measurement instruments to identify patient changes based on behavioural cues, HCPs may find it challenging to interpret and use scores and determine which multimodal interventions to use. The overlap among similar behavioural cue items across measurement instruments and the multiple measurement instruments available has proved challenging for HCPs.^13 14^ Pain and sedation, and delirium and withdrawal, are concomitant pairs. This is demonstrated by the development and use of measurement instruments for these pairs (eg, COMFORT behaviour scale^43^ and SOS-PD^44^). Using measurement instruments is the first step towards goal-directed care, and this review will synthesise strategies to inform clinical practice.

Based on quality appraisal, the results will establish which CPGs can be recommended for use and implementation into clinical practice. It will also provide accessible summaries of the best evidence for each recommendation and type of care intervention for the four conditions to support implementation into practice. These results can be used as the basis for the development of a combined CPG for these four conditions specific for the PICU.

The strengths of this systematic review are the comprehensive search for CPGs on the four interrelated conditions, which has not been previously conducted, as well as the evaluation of the quality of CPGs and the base of evidence for included recommendations.

A limitation of this systematic review will be ensuring the review team is sufficiently trained in using the AGREE-REX, as this is a novel tool. Currently, no training resources exist and guidance on its use during the systematic review process is lacking. This might lead to divergent scores. To mitigate this limitation, a member of the review team will develop a training video, and the researchers will use a consensus process. It is likely that the heterogeneity of research conducted in the PICU setting will lead to low-quality scores for recommendations.

## Conclusion

Management of pain and sedation is a balancing act for HCPs in order to provide optimal comfort and avoid delirium and iatrogenic withdrawal for their paediatric patients. Recommendations for managing these four interrelated conditions are mixed. This systematic review will use rigorous methods to assess the quality and content of CPGs and included recommendations for the assessment and management of these four conditions. It will add to the current body of knowledge with the intention to optimise care and outcomes for critically ill paediatric patients in the PICU.

## Supplementary Material

Author's manuscript

## Data Availability

Data sharing not applicable as no datasets generated and/or analysed for this study. No data are available.
