# Supplementary material for: Quality of clinical practice guidelines and recommendations for the management of pain, sedation, delirium and iatrogenic withdrawal in pediatric intensive care: a systematic review protocol
Source: BMJ Paediatr Open. 2022 Feb 15;6(1):e001293. doi: 10.1136/bmjpo-2021-001293 (PMC8852722; doi:10.1136/bmjpo-2021-001293)
Supplement: Supplementary data [file bmjpo-2021-001293supp001.pdf]

SUPPLEMENTARY MATERIALS

Table 1A: PRISMA-P[1] (Preferred Reporting Items for Systematic review and Meta-Analysis Protocols) 2015 checklist: recommended items to address in a systematic review protocol

Section and topic	Item No	Checklist item	Page number (line)
**ADMINISTRATIVE INFORMATION**			
Title:			
Identification	1a	Identify the report as a protocol of a systematic review	6 (105 - 112) + title
Update	1b	If the protocol is for an update of a previous systematic review, identify as such	N/A
Registration	2	If registered, provide the name of the registry (such as PROSPERO) and registration number	Abstract + 6 (108-112)
Authors:			
Contact	3a	Provide name, institutional affiliation, e-mail address of all protocol authors; provide physical mailing address of corresponding author	1 (3-5 +table)
Contributions	3b	Describe contributions of protocol authors and identify the guarantor of the review	12 (270 – 273)
Amendments	4	If the protocol represents an amendment of a previously completed or published protocol, identify as such and list changes; otherwise, state plan for documenting important protocol amendments	6 (111-112)
Support:			
Sources	5a	Indicate sources of financial or other support for the review	12 (275-276)
Sponsor	5b	Provide name for the review funder and/or sponsor	N/A
Role of sponsor or funder	5c	Describe roles of funder(s), sponsor(s), and/or institution(s), if any, in developing the protocol	N/A
**INTRODUCTION**			
Rationale	6	Describe the rationale for the review in the context of what is already known	4-5 (36 -95)
Objectives	7	Provide an explicit statement of the question(s) the review will address with reference to participants, interventions, comparators, and outcomes (PICO)	5-6 (95-103)
**METHODS**			
Eligibility criteria	8	Specify the study characteristics (such as PICO, study design, setting, time frame) and report characteristics (such as years considered, language, publication status) to be used as criteria for eligibility for the review	6-7 (113-128; Table 1: PICAR)
Information sources	9	Describe all intended information sources (such as electronic databases, contact with study authors, trial registers or other grey literature sources) with planned dates of coverage	8 (132 – 139) (supplement table 1B)

Search strategy	10	Present draft of search strategy to be used for at least one electronic database, including planned limits, such that it could be repeated	8 (140-146) (supplement table 1C)
Study records:			
Data management	11a	Describe the mechanism(s) that will be used to manage records and data throughout the review	8 (148 – 150)
Selection process	11b	State the process that will be used for selecting studies (such as two independent reviewers) through each phase of the review (that is, screening, eligibility and inclusion in meta-analysis)	8 (150 – 157)
Data collection process	11c	Describe planned method of extracting data from reports (such as piloting forms, done independently, in duplicate), any processes for obtaining and confirming data from investigators	9 (161 – 164)
Data items	12	List and define all variables for which data will be sought (such as PICO items, funding sources), any pre-planned data assumptions and simplifications	9 (165 – 174) (supplement tables 1D – 4D)
Outcomes and prioritization	13	List and define all outcomes for which data will be sought, including prioritization of main and additional outcomes, with rationale	9 - 10 (190-193)
Risk of bias in individual studies	14	Describe anticipated methods for assessing risk of bias of individual studies, including whether this will be done at the outcome or study level, or both; state how this information will be used in data synthesis	9 (176-189)
Data synthesis	15a	Describe criteria under which study data will be quantitatively synthesized	N/A
	15b	If data are appropriate for quantitative synthesis, describe planned summary measures, methods of handling data and methods of combining data from studies, including any planned exploration of consistency (such as I^2, Kendall's τ)	N/A
	15c	Describe any proposed additional analyses (such as sensitivity or subgroup analyses, meta-regression)	N/A
	15d	If quantitative synthesis is not appropriate, describe the type of summary planned	10-11 (216 – 226)
Meta-bias(es)	16	Specify any planned assessment of meta-bias(es) (such as publication bias across studies, selective reporting within studies)	None
Confidence in cumulative evidence	17	Describe how the strength of the body of evidence will be assessed (such as GRADE)	11 (205 - 215)

N/A = not applicable

[1] Moher D, Shamseer L, Clarke M, et al. Preferred reporting items for systematic review and meta-analysis protocols (PRISMA-P) 2015 statement. *Systematic reviews* 2015;4(1):1.

Table 1B: Guideline repositories and Professional Societies/Organizations

	Country/Region	Website
**Guideline repositories**		
Australian Clinical Practice Guidelines Portal	Australia	https://www.clinicalguidelines.gov.au/portal
BIGG International database of GRADE guidelines	Worldwide	https://sites.bvsalud.org/bigg/en/biblio/
CISMeF Bonnes pratiques	France	https://doccismef.chu-rouen.fr/dc/#env=bp
ECRI Guidelines Trust	USA/UK	https://guidelines.ecri.org/
Guideline Central	USA	https://www.guidelinecentral.com/summaries/#summary-view-specialty
Guidelines International Network (G-I-N)	Scotland	https://guidelines.ebmportal.com/
Infobanque Guides de Pratiques Clinique (GPC)	Canada	https://jouleamc.ca/cpg/homepage
NICE Evidence Search	UK	https://www.evidence.nhs.uk/
TRIP	UK	https://www.tripdatabase.com/
Up-to-date Society guideline links	Netherlands	http://www.uptodate.com/contents/search
**Professional societies/organizations**		
American Association of Critical-care Nurses	USA	https://www.aacn.org/clinical-resources/view-all-issues?category=practice-alerts
European Society of Intensive Care Medicine (ESICM)	Europe	https://www.esicm.org/

European Society of Paediatric Neonatal Intensive Care (ESPNIC)	Europe	https://espnic-online.org
National Institute for Health and Care Excellence (NICE)	UK	www.nice.org.uk
Registered Nurses' Association of Ontario (RNAO)	Canada	https://rnao.ca/bpg
Royal College of Nursing	UK	https://www.rcn.org.uk/
Scottish Intercollegiate Guidelines Network (SIGN)	Scotland	http://sign.ac.uk/index.html
Société de Réanimation de Langue Française (SRLF)	France	https://srlf.org
Société Française d'Anesthésie et de Réanimation (SFAR)	France	https://sfar.org
Société Suisse de Pédiatrie	Switzerland	https://www.paediatrieschweiz.ch/fr/
Société Suisse de Médecine Intensive (SSMI)	Switzerland	https://www.sgi-ssmi.ch/fr
Society of Critical Care Medicine (SCCM)	USA	https://www.sccm.org/Home
World Federation of Pediatric Intensive and Critical Care Societies (WFPICCS)	Worldwide	httsp://wfpiccs.org

Table 1C: Final search strategy for Embase.com

('pain'/de OR 'postoperative pain'/de OR 'analgesia'/exp OR 'conscious sedation'/de OR 'hypnotic sedative agent'/de OR 'delirium'/de OR 'hyperactive delirium'/de OR 'hypoactive delirium'/de OR 'withdrawal syndrome'/de OR (pain OR discomfort OR analgesia OR sedation OR sedative* OR delirium OR delirious* OR withdrawal*):ti,kw) AND ('practice guideline'/de OR 'consensus development'/de OR ((expert* NEAR/3 opinion*) OR guideline* OR cpg* OR guidance OR ((position OR policy) NEAR/3 (paper* OR development)) OR ((practice OR clinical) NEAR/3 development) OR (practice NEAR/2 guide$) OR recommendation* OR consensus OR standards OR statement*):ti,kw) NOT ('adult'/exp NOT 'juvenile'/exp) AND [2010-3000]/py NOT ('conference abstract'/it OR 'conference review'/it) NOT ([animals]/lim NOT [humans]/lim)

9849 references (17 November 2021)

Figures 1D – 4D: Figures of excel spreadsheets for data collection

Figure 1D: General Information

Title	First Author	Publication year	Language	Developing / publishing organisation and / or authors	Country / region of publication	How described by the authors (e.g. guideline / consensus statement / position stateme	Version	Topic addressed (pain) [insert 1]	Topic addressed (sedation) [insert 1]	Topic addressed (delirium) [insert 1]	Topic addressed (withdrawal) [insert 1]	Target population (A+adult, C=child, NC=non-communicative)[insert all that apply)	Target setting (G=general; S=specific) [specify e.g. G (acute care) S (PICU)	LoE rating system	GoE rating system

Figure 2D: AGREE II

	CPG#1 [Name]		CPG#2 [Name]		CPG
**Domain 1: Scope and purpose**	Reviewer 1	Reviewer2	Reviewer 1	Reviewer2	Review
1. The overall objective(s) of the guideline is (are) specifically described					
2. The health question(s) covered by the guideline is (are) specifically described					
3. The population (patients, public, etc.) to whom the guideline is meant to apply is specifically described					
**Domain 2: Stakeholder involvement**					
4. The guideline development group includes individuals from all relevant professional groups					
5. The views and preferences of the target population (patients, public, etc.) have been sought					
6. The target users of the guideline are clearly defined					
**Domain 3: Rigour of development**					
7. Systematic methods were used to search for evidence					
8. The criteria for selecting the evidence are clearly described					
9. The strengths and limitations of the body of evidence are clearly described					
10. The methods for formulating the recommendations are clearly described					
11. The health benefits, side effects, and risks have been considered in formulating the recommendations					
12. There is an explicit link between the recommendations and the supporting evidence					
13. The guideline has been externally reviewed by experts prior to its publication					
14. A procedure for updating the guideline is provided					
**Domain 4: Clarity of presentation**					
15. The recommendations are specific and unambiguous					
16. The different options for management of the condition or health issue are clearly presented					
17. Key recommendations are easily identifiable					
**Domain 5: Applicability**					
18. The guideline describes facilitators and barriers to its application					
19. The guideline provides advice and/or tools on how the recommendations can be put into practice					
20. The potential resource implications of applying the recommendations have been considered					
21. The guideline presents monitoring and/or auditing criteria					
**Domain 6: Editorial independence**					
22. The views of the funding body have not influenced the content of the guideline					
23. Competing interests of guideline development group members have been recorded and addressed					
**Overall guideline assessment**					
1. Rate the overall quality of this guideline					

Figure 3D: AGREE-REX

Guideline Name	CPG#1 [Name]		CPG#2 [Name]		CPG#3 [Na	
**Domain 1: Clinical Applicability**	Reviewer 1	Reviewer2	Reviewer 1	Reviewer2	Reviewer 1	Rev
1. Evidence						
2. Applicability to Target Users						
3. Applicability to Patients/Populations						
**Domain 2: Values and Preferences**						
4. Values and Preferences of Target Users						
5. Values and Preferences of Patients/Populations						
6. Values and Preferences of Policy/Decision-Makers						
7. Values and Preferences of Guideline Developers						
**Domain 3: Implementability**						
8. Purpose						
9. Local Application and Adoption						
1. I would recommend these guideline recommendations for use in the appropriate context.						
2. I would recommend these guideline recommendations for use in my context (optional).						

Figure 4D: Recommendations and evidence (one table per concept)

Guideline Title	Recommendation	Grade of Recommendation	Evidence cited for recommendation (Author, year)	Recommendation category specified within guideline
