# Supplementary material for: Quality of clinical practice guidelines and recommendations for the management of pain, sedation, delirium and iatrogenic withdrawal in pediatric intensive care: a systematic review protocol
Source: BMJ Paediatr Open. 2022 Feb 15;6(1):e001293. doi: 10.1136/bmjpo-2021-001293 (PMC8852722; doi:10.1136/bmjpo-2021-001293)
Supplement: Author's manuscript [file bmjpo-2021-001293.draft_revisions.pdf]

BMJ Paediatrics Open

BMJ Paediatrics Open is committed to open peer review. As part of this commitment we make the peer review history of every article we publish publicly available.

When an article is published we post the peer reviewers' comments and the authors' responses online. We also post the versions of the paper that were used during peer review. These are the versions that the peer review comments apply to.

The versions of the paper that follow are the versions that were submitted during the peer review process. They are not the versions of record or the final published versions. They should not be cited or distributed as the published version of this manuscript.

BMJ Paediatrics Open is an open access journal and the full, final, typeset and author-corrected version of record of the manuscript is available on our site with no access controls, subscription charges or pay-per-view fees (http://bmjpaedsopen.bmj.com).

If you have any questions on BMJ Paediatrics Open's open peer review process please email info.bmjpo@bmj.com

BMJ Paediatrics Open

Quality of clinical practice guidelines and recommendations for the management of pain, sedation, delirium, and withdrawal in pediatric intensive care: A systematic review protocol

Journal:	*BMJ Paediatrics Open*
Manuscript ID	bmjpo-2021-001293
Article Type:	Protocol
Date Submitted by the Author:	10-Sep-2021
Complete List of Authors:	MacDonald, Ibo; University of Lausanne, University Institute of Higher Education and Research in Healthcare<br>Perez, Marie-Hélène; Lausanne University Hospital Department of Woman Mother and Child<br>Trombert, Alexia; Lausanne University Hospital, Medical Library<br>Amiet, Vivianne; Lausanne University Hospital Department of Woman Mother and Child<br>Ramelet, Anne-Sylvie; University of Lausanne Faculty of Biology and Medicine, University Institute of Higher Education and Research in Healthcare
Keywords:	Pain

SCHOLARONE™
Manuscripts

I, the Submitting Author has the right to grant and does grant on behalf of all authors of the Work (as defined in the below author licence), an exclusive licence and/or a non-exclusive licence for contributions from authors who are: i) UK Crown employees; ii) where BMJ has agreed a CC-BY licence shall apply, and/or iii) in accordance with the terms applicable for US Federal Government officers or employees acting as part of their official duties; on a worldwide, perpetual, irrevocable, royalty-free basis to BMJ Publishing Group Ltd ("BMJ") its licensees and where the relevant Journal is co-owned by BMJ to the co-owners of the Journal, to publish the Work in this journal and any other BMJ products and to exploit all rights, as set out in our licence.

The Submitting Author accepts and understands that any supply made under these terms is made by BMJ to the Submitting Author unless you are acting as an employee on behalf of your employer or a postgraduate student of an affiliated institution which is paying any applicable article publishing charge ("APC") for Open Access articles. Where the Submitting Author wishes to make the Work available on an Open Access basis (and intends to pay the relevant APC), the terms of reuse of such Open Access shall be governed by a Creative Commons licence – details of these licences and which Creative Commons licence will apply to this Work are set out in our licence referred to above.

Other than as permitted in any relevant BMJ Author's Self Archiving Policies, I confirm this Work has not been accepted for publication elsewhere, is not being considered for publication elsewhere and does not duplicate material already published. I confirm all authors consent to publication of this Work and authorise the granting of this licence.

Confidential: For Review Only

Quality of clinical practice guidelines and recommendations for the management of pain, sedation, delirium, and withdrawal in pediatric intensive care: Systematic review protocol

Ibo MacDonald[1], Marie-Hélène Perez[2], Vivianne Amiet[2], Alexia Trombert[3], Anne-Sylvie Ramelet[1,2]*

Full name	Institution, Department	Postal address		e-mail	ORCID ID
*Corresponding author					
Anne-Sylvie Ramelet	University Institute of Higher Education and Research in Healthcare, Faculty of Biology and Medicine, University of Lausanne and Department Woman-Mother-Child, Lausanne University Hospital (CHUV),	Route de la Corniche 10, Lausanne, Switzerland, CH-1010		Anne-sylvie.ramelet@chuv.ch	0000-0001-8809-2920
Co-authors	Institution, Department	City	Country	e-mail	
Ibo MacDonald	University Institute of Higher Education and Research in Healthcare, Faculty of Biology and Medicine, University of Lausanne	Lausanne	Switzerland	Ibo.macdonald@chuv.ch	0000-0001-9490-8850
Marie-Hélène Perez	Lausanne University Hospital, Department Woman-Mother-Child and University of Lausanne			Marie-helene.perez@chuv.ch	0000-0001-6173-3396
Vivianne Amiet	Lausanne University Hospital, Department Woman-Mother-Child and University of Lausanne			Vivianne.Amiet@chuv.ch	0000-0002-6665-5291
Alexia Trombert	Lausanne University Hospital and University of Lausanne, Medical Library			Alexia.Trombert@chuv.ch	0000-0002-4833-5219

SUMMARY
Word count: 2133/2500
Abstract: 298/300
Tables/figures: 1/8
References: 40/40

Confidential: For Review Only

ABSTRACT (298/300)

Introduction: Inadequate management of pain and sedation in critically ill children can cause unnecessary suffering and agitation, but also delirium and withdrawal. It is therefore important to address these four interrelated symptoms together. Some clinical practice guidelines (CPGs) are available for the management of pain and sedation, and a few for delirium and withdrawal in the PICU; none address the four symptoms altogether. Critical appraisal of the quality of CPGs is necessary for their recommendations to be adopted into clinical practice. The aim of this systematic review is to identify and appraise the quality of CPGs and recommendations for management of either pain, sedation, delirium and withdrawal.

Methods and analysis: A systematic review will be conducted in electronic databases (Medline ALL (Ovid), Embase.com, CINAHL (EBSCO), Joanna Briggs Institute (JBI) EBP Database), guideline repositories and websites of professional societies to identify CPGs published from 2010 to date in English and French languages. Index and free terms describing CPGs will be combined with pain, sedation, delirium and withdrawal. CPGs will be included if they can be applied in the pediatric critical care population (newborns to 18 years old) and include recommendation(s) for assessment of at least one of the four symptoms. Two independent reviewers will screen for eligibility, complete data extraction and quality assessment using the Appraisal of Guidelines for Research and Evaluation (AGREE) II and the AGREE Recommendation Excellence (AGREE-REX) instruments. Characteristics, content and recommendations from CPGs will be reported in tabulated forms.

Ethics and dissemination: Ethical approval is not required for this systematic review. Results will be published in a peer-reviewed journal.

Trial registration: This protocol has been submitted for registration to the international database of prospectively registered systematic reviews (PROSPERO, awaiting registration number).

Key words: AGREE II, children, clinical practice guideline, delirium, pain, sedation, systematic review, withdrawal

Confidential: For Review Only

SUMMARIZING BOX

What is known about the subject (2/3 statements)

- Optimal analgesia and sedation management is challenging in the pediatric intensive care unit. (13/25 words)
- Pain, sedation, delirium and withdrawal are interrelated symptoms that need to be assessed as a whole to prevent negative outcomes. (20/25 words)

What this study hopes to add (2/3 statements)

- This review will compare the quality of CPGs across all four interrelated symptoms of pain, sedation, delirium and withdrawal. (19/25 words)
- This review will compare recommendations by describing the certainty and applicability of the base of evidence for CPGs. (18/25 words)

Confidential: For Review Only

INTRODUCTION

Critically ill children in the pediatric intensive care unit (PICU), particularly those who are mechanically ventilated, require adequate identification and treatment of pain, sedation, delirium and withdrawal.[1] Management involves the use of analgesics and sedatives, often in combination, with 65% of children receiving both.[2] Optimal titration of analgesics and sedatives prevent on one hand pain, delirium, withdrawal,[3,4] and on the other hand accidental extubation.[5] Over sedation and analgesia can result in prolonged mechanical ventilation, increased PICU length of stay, morbidity and mortality.[1] In the PICU setting 40-65% of children are unable to self-report because of being mechanically ventilated and also due to their young age (under the age of four).[6,7] Given the large proportion of non-communicative children, the use of multiple measurement instruments to assess pain, sedation, delirium and withdrawal are crucial for quality of care.

Many measurement instruments have been developed for use by healthcare professionals (HCPs) for pain and sedation,[8] and more recently, for delirium,[9] and withdrawal.[10] These measurement instruments incorporate items for behavioural cues, however, many of these items overlap amongst the four symptoms and within measurement instruments.[11,12] International survey studies on the utilization of instruments for these four symptoms in PICUs have shown wide variation in their availability and application into standardized care practices, such that protocols and algorithms with measurement instruments incorporated are infrequently used.[13-16] Consequently, there has been a call from researchers to avoid looking at these symptoms in siloes and to incorporate them together into standardized care practices,[11,12] either in clinical practice guidelines (CPGs) or as protocols or algorithms to improve assessment and management.

CPGs are created by synthesizing research to help bridge the evidence-to-practice gap. The purported benefits of CPG implementation are the standardization of care practices, improvements in patient safety and patient outcomes.[17] Few CPGs have been developed for management of these four symptoms in the PICU with only two for pain,[18,19] and one for pain, sedation and delirium.[20] Furthermore, some guidance documents exist (these include practice/position/consensus statements/recommendations (hereafter, referred to as CPGs)).[11,21,22] Although, there is one position statement for the assessment of pain, sedation, delirium and withdrawal for pediatric critical care patients,[11] it does not include recommendations for management. Previous systematic reviews of CPGs for pain in pediatrics

Confidential: For Review Only

exist, they target neonates,[23] or burn patients,[24] or focus on procedural[23,25] or acute pain.[24] Systematic reviews of CPGs concerning best practices for children in the PICU are lacking, to date, none have been conducted on either of the four symptoms, nor have they been examined together. Recently, scholars have criticized systematic reviews of CPGs and their lack of quality appraisal of recommendations.[26] Several systematic reviews of CPG recommendations have demonstrated evidence that weakly supports the recommendations.[27,28] A recent review of CPGs for pediatric populations published between 2017 to 2019 found that 75% of the 216 CPGs were evidence-based.[29] Establishing methodological quality of CPGs and the evidence base of recommendations is important for promoting evidence-informed interventions.

Quality CPGs and their contained recommendations should reflect the most current evidence. Although, numerous appraisal instruments exist for assessing the quality of CPGs.[30] Internationally, the appraisal of guidelines for research and evaluation (AGREE) II has emerged as the most widely used appraisal instrument.[31] The AGREE Enterprise recently developed the AGREE recommendation of excellence (AGREE-REX) instrument for the quality appraisal of recommendations.[32] Thus, jointly using these two instruments allows for assessing CPG quality and for ensuring that the evidence supporting recommendations in CPGs are reliable and trustworthy. This is essential for CPGs to remain sources of information that clinicians use to improve their practice and care of patients. The identification, appraisal and comparison of quality of CPGs and their recommendations is a valuable first step in informing efforts to incorporate these four overlapping symptoms together in a standardized way to optimize care in the PICU. The aim of this systematic review is to appraise the quality of CPGs and recommendations for the management of pain, sedation, delirium and withdrawal in the PICU. The specific objectives are:

1. To identify published CPGs for the management of pain, sedation, delirium and withdrawal;
2. To appraise the quality of selected CPGs;
3. To appraise the quality of recommendations included in CPGs; and
4. To summarize the convergence of recommendations and the overall robustness of recommendation in CPGs.

METHODS AND ANALYSIS

Confidential: For Review Only

This study protocol used the methodological guide for conducting systematic reviews of CPGs to guide the development of each stage.[33] This study protocol is reported according to the preferred reporting items for systematic review and meta-analysis protocols (PRISMA-P) (online supplemental material appendix A).[34] The study protocol was submitted for registration to the international database of prospectively registered systematic reviews (PROSPERO, awaiting registration number). In the event of amendment to this protocol, the PROSPERO register will be updated.

Inclusion/exclusion criteria for study selection

To guide CPG selection the population, intervention, comparators, attributes and recommendations (PICAR) framework was used (see Table 1).[33] For the purpose of this review, CPGs must include recommendations developed from available evidence, including expert opinion.[17] CPGs and guidance statements will be considered. CPGs which include pediatric populations will be included in this review if they: i) are endorsed by a society, ii) include a recommendation for assessment of any of the four symptoms, and iii) are the most current version. Restrictions will be applied to publication year from 2010 to present and to language, including only English and French, as these are the languages of the review team.

Table 1: PICAR statement: inclusion and exclusion criteria

**P**opulation, clinical indication(s), and condition(s)	**Study population**:<br>- Include: Children (newborn (>38 weeks gestations) to 18 years of age)<br>- Exclude : premature infants<br>**Clinical indications**:<br>- Include: management of either pain, sedation, delirium ot withdrawal<br>- Exclude: Management specific to medications<br>**Conditions**:<br>- Include: children in intensive care, with a focus on non-communicative
**I**nterventions	Any intervention focusing on management of either pain, sedation, delirium or withdrawal
**C**omparator(s), comparison(s), and (key) content	**Comparator/comparison:** Any<br>**Key content**:<br>- If a broad population is included, the CPG must have separate recommendations for children<br>- The CPG can be implemented in the intensive care setting but does not need to be specifically developed for intensive care<br>- The CPG must include recommendations on assessment for either pain, sedation, delirium or withdrawal
**A**ttributes of eligible CPGs	**Language:** English and French<br>**Year of publication**: 2010 onward

Confidential: For Review Only

	**Setting**:<br>- Include: Applicable to pediatric intensive care, can be broad/general<br>- Exclude: CPGs developed specifically for other settings: neonatal intensive care units, emergency department, pre-operative/operating room<br>**Developing/publishing organization:**<br>- Included: CPGs issued or endorsed by international, national or regional societies/professional organizations or governments from developed countries.<br>- Exclude: CPGs that were developed by an individual organization (e.g. hospital) or unit within an organization.<br>**Version**: Latest/newest version<br>**Type**: CPGs, consensus statements, practice/position recommendations/alerts/ statements<br>**Quality score**: The AGREE II will be used to assess quality but will not be used as an eligibility criteria for inclusion in the systematic review.
**R**ecommendations characteristics and “other” considerations	**Recommendations:** CPGs must have a specific recommendation for assessment of either pain, sedation, delirium or withdrawal (either explicitly highlighted as a recommendation (primary) or noted in the body of the text (secondary - not explicitly identified as a recommendation)

Search methods

Informational sources

The search will be conducted in:

1) Four electronic databases: Medline ALL (Ovid), Embase.com, CINAHL (EBSCO) and Joanna Briggs Institute (JBI) EBP Database.
2) Six guideline repositories.
3) Fourteen professional societies/organizations (online supplemental material appendix B contains a list of guideline repositories and professional societies/organizations to be searched).

Search strategy

The search strategy will be developed with the assistance of a health services librarian. Index and free terms describing CPGs and pain, sedation, delirium, withdrawal will be combined to create an advanced search strategy that will be translated for all databases and sources of information. A preliminary search strategy for Embase.com is provided in the online

Confidential: For Review Only

supplemental material appendix C. The search strategy will be peer reviewed by another librarian using the PRESS checklist.[35]

Guideline selection

The search results will be imported into Endnote X9 reference manager (Clarivate Analytics, USA) for duplicate removal. The remaining citations will be uploaded to Rayyan QCRI (Qatar Computing Research Institute, Doha, Qatar) to manage the screening process.[36]

Two independent reviewers will screen titles and abstracts of all citations to determine those for full-text review which will be retrieved and assessed against the inclusion and exclusion criteria. Reasons for exclusion will be recorded. Any disagreements will be resolved through discussion and consensus or by a third reviewer.

To ensure all relevant documents are available for quality appraisal for included CPGs, the review team will visit the endorsing organizations website (if available) to retrieve supporting documents (e.g. evidence tables, conflict of interest declarations).

The PRISMA flow diagram will be used to show the selection process and summarise the inclusion and exclusion details.[37]

Data extraction

Information from each included CPGs will be extracted by two independent reviewers. The review team developed an Excel spreadsheet for data extraction (online supplemental material appendix D) that will be piloted and revised during the data extraction phase. The following key areas will be extracted:

a) General information: title, first author, year of publication, language, developing organization, country, type of CPG, topic addressed (pain, sedation, delirium, withdrawal), target population, target setting, level of evidence (LoE) rating system, grade of evidence (GoE) rating system.
b) Quality of included CPGs using the AGREE II instrument (details below).[31]
c) Quality of recommendations from CPGs using the AGREE-REX instrument (details below).[32]

Confidential: For Review Only

d) Recommendations (one worksheet per symptom and each line will represent one recommendation): recommendation, grade of recommendations, classification of evidence, list supporting citation(s), categorization of recommendation as per CPG.

Quality appraisal of CPGs and recommendations

Quality of CPGs. At least two reviewers will independently appraise each included CPG using the AGREE II instrument.[31] The AGREE II is a validated and reliable appraisal instrument for assessing the quality of CPGs.[31] It contains 23 items across six domains: 1) scope and purpose; 2) stakeholder involvement; 3) rigour of development; 4) clarity of presentation; 5) applicability; and 6) editorial independence. Reviewers appraise each item against a 7-point Likert scale ranging from 1 (strongly disagree) to 7 (strongly agree). In addition, there are two global rating scores: 1) overall quality of the CPG, and 2) whether the guideline would be recommended for use.

Quality of CPGs recommendations. The AGREE–REX will be used for assessing the quality of recommendations.[32] It is a recently developed, valid and reliable appraisal instrument containing 9 items across three domains: 1) clinical applicability; 2) values and preferences; and 3) implementability.[32] Two reviewers will appraise each item using the same 7-point Likert scale as the AGREE II. It includes a global rating score of the overall quality of the CPGs recommendations.

The same scoring is used for both the AGREE II and the AGREE-REX, whereby, each items' score across reviewers is summed and converted to a percentage of the maximum possible score for each domain.[31]

To ensure standardization of appraisal each reviewer will be trained using the training tools available on the AGREE II website (www.agreetrust.com). Inter-rater agreement will be calculated using intra-class correlation coefficients (ICCs) with a two-way random effects model for each domain. The levels of ICC agreement will be classified as poor (<0.50), moderate (0.50–0.75), good (0.75–0.9), and excellent (>0.9).[38]

Data synthesis

Quality of CPGs and recommendations: The AGREE Enterprise has no established quality threshold, instead review teams must establish their own prior to appraisal.[39] As recommended in a recent systematic review of AGREE II thresholds for determining CPG

Confidential: For Review Only

quality, we will use the 3-step system where high quality are scores > 60%, medium quality are scores between 60% and 30%, and low quality are < 30% across all domains.[40]

The results of the AGREE II and AGREE-REX scores will be presented in a table. The quality of each domain will be presented as a heat map based on the same threshold cut-off as for quality of CPG as described above.

Synthesis of recommendations and their level of evidence: All recommendations and their related evidence will be extracted per symptom. Once extracted each recommendation will be categorized based on the type of care intervention, including: a) prevention, b) assessment, and c) management. Management interventions will be further divided into pharmacological and/or non-pharmacological. Summary tables will be created to highlight the consistency of all recommendations for each symptom. We will report the LoE associated with recommendations within each CPG but we will not attempt to standardize them across CPGs.

ETHICS AND DISSEMINATION

Ethical approval is not required for this systematic review. To disseminate the results the study will be published in a peer-reviewed journal.

PATIENT AND PUBLIC INVOLVEMENT

There was no patient or public involvement in the development of the systematic review protocol. The Swiss Society of Intensive Care Medicines' Pain, Agitation, Delirium, Immobility and Sleep (PADIS-CH) working group will be involved in data synthesis as clinical experts.

DISCUSSION

This systematic review of CPGs will be a valuable first step in informing efforts in standardizing the management of pain, sedation, delirium and withdrawal in the PICU. Based on quality appraisal, the results will establish which CPGs can be recommended for use in clinical practice. It will also provide accessible summaries of the best evidence for each recommendation and type of care intervention for the four symptoms. This will in turn help to identify the gaps for recommendations, where inconsistencies exist and where high quality evidence is lacking to support implementation into practice.

Confidential: For Review Only

The strengths of this systematic review is the comprehensive search for CPGs on the four interrelated symptoms which has not been previously conducted as well as the evaluation of the quality of the base of evidence for each recommendation.

A limitation of this systematic review is despite a comprehensive search strategy, the restriction to publications in English or French may exclude relevant CPGs published in other languages.

CONCLUSION

Management of pain and sedation is a balancing act to provide optimal comfort and avoid delirium and withdrawal. Recommendations for managing these four interrelated symptoms are mixed. This systematic review will use rigorous methods to assess the quality and content of CPGs and included recommendations for the management of these four symptoms. It will add to the current body of knowledge with the intention to optimise care and outcomes for critically ill pediatric patients in the PICU.

AUTHOR CONTRIBUTIONS. The conception and design of this systematic review protocol was completed by three authors (IMD, ASR, MHP). The search strategy was developed by IMD and AT with feedback from the review team (ASR, MHP and VA). IMD prepared the first draft of the manuscript with all authors providing feedback towards the final manuscript.

FUNDING STATEMENT. This research received no specific grant from any funding agency in the public, commercial or not-for-profit sectors.

COMPETING INTERESTES. None declared

REFERENCES

1. Egbuta C, Mason KP. Current State of Analgesia and Sedation in the Pediatric Intensive Care Unit. *J Clin Med* 2021;10(9):1847. doi: 10.3390/jcm10091847 [published Online First: 2021/05/01]
2. Patel AK, Trujillo-Rivera E, Faruqe F, et al. Sedation, Analgesia, and Neuromuscular Blockade: An Assessment of Practices From 2009 to 2016 in a National Sample of 66,443 Pediatric Patients Cared for in the ICU. *Pediatr Crit Care Med* 2020;21(9):e599-e609. doi: 10.1097/PCC.0000000000002351 [published Online First: 2020/03/21]
3. Best KM, Boullata JI, Curley MA. Risk factors associated with iatrogenic opioid and benzodiazepine withdrawal in critically ill pediatric patients: a systematic review and conceptual model. *Pediatr Crit Care Med* 2015;16(2):175-83. doi: 10.1097/PCC.0000000000000306 [published Online First: 2015/01/07]
4. Mody K, Kaur S, Mauer EA, et al. Benzodiazepines and Development of Delirium in Critically Ill Children: Estimating the Causal Effect. *Crit Care Med* 2018;46(9):1486-91. doi: 10.1097/CCM.0000000000003194 [published Online First: 2018/05/05]

Confidential: For Review Only

5. Lucas da Silva PS, de Carvalho WB. Unplanned extubation in pediatric critically ill patients: a systematic review and best practice recommendations. *Pediatr Crit Care Med* 2010;11(2):287-94. doi: 10.1097/PCC.0b013e3181b80951 [published Online First: 2009/10/02]
6. Ibiebele I, Algert CS, Bowen JR, et al. Pediatric admissions that include intensive care: a population-based study. *BMC Health Serv Res* 2018;18(1):264. doi: 10.1186/s12913-018-3041-x [published Online First: 2018/04/11]
7. Laures E, LaFond C, Hanrahan K, et al. Pain Assessment Practices in the Pediatric Intensive Care Unit. *J Pediatr Nurs* 2019;48:55-62. doi: 10.1016/j.pedn.2019.07.005 [published Online First: 2019/07/22]
8. Giordano V, Edobor J, Deindl P, et al. Pain and Sedation Scales for Neonatal and Pediatric Patients in a Preverbal Stage of Development: A Systematic Review. *JAMA Pediatr* 2019;173(12):1186-97. doi: 10.1001/jamapediatrics.2019.3351 [published Online First: 2019/10/15]
9. Smith HA, Gangopadhyay M, Goben CM, et al. The Preschool Confusion Assessment Method for the ICU: Valid and Reliable Delirium Monitoring for Critically Ill Infants and Children. *Crit Care Med* 2016;44(3):592-600. doi: 10.1097/CCM.0000000000001428 [published Online First: 2015/11/14]
10. Ista E, de Hoog M, Tibboel D, et al. Psychometric evaluation of the Sophia Observation withdrawal symptoms scale in critically ill children. *Pediatr Crit Care Med* 2013;14(8):761-9.doi: 10.1097/PCC.0b013e31829f5be1 [published Online First: 2013/08/22]
11. Harris J, Ramelet AS, van Dijk M, et al. Clinical recommendations for pain, sedation, withdrawal and delirium assessment in critically ill infants and children: an ESPNIC position statement for healthcare professionals. *Intensive Care Med* 2016;42(6):972-86. doi: 10.1007/s00134-016-4344-1 [published Online First: 2016/04/17]
12. Ista E, van Dijk M. We can not compartmentalize our patients! Overlapping symptoms of iatrogenic withdrawal syndrome, pediatric delirium, and anticholinergic toxidrome. *Pediatric critical care medicine* 2017;18(6):603-04.doi: 10.1097/PCC.0000000000001163
13. Tabacco B, Tacconi C, Amigoni A. Survey on monitoring analgesia and sedation in the Italian Pediatric Intensive Care Units. *Minerva Anestesiol* 2017;83(10):1010-16. doi: 10.23736/S0375-9393.17.11707-4 [published Online First: 2017/03/31]
14. Staveski SL, Pickler RH, Lin L, et al. Management of pediatric delirium in pediatric cardiac intensive care patients: An international survey of current practices. *Pediatric Critical Care Medicine* 2018;19(6):538-43. doi: 10.1097/PCC.0000000000001558
15. Koizumi T, Kurosawa H. Survey of analgesia and sedation in pediatric intensive care units in Japan. *Pediatr Int* 2020;62(5):535-41. doi: 10.1111/ped.14139 [published Online First: 2020/01/08]
16. Kudchadkar SR, Yaster M, Punjabi NM. Sedation, sleep promotion, and delirium screening practices in the care of mechanically ventilated children: a wake-up call for the pediatric critical care community*. *Crit Care Med* 2014;42(7):1592-600. doi: 10.1097/CCM.0000000000000326 [published Online First: 2014/04/11]
17. Graham R. Clinical practice guidelines we can trust. Washington, DC: National Academies Press 2011.
18. Association of Paediatric Anaesthetists of Great B, Ireland. Good practice in postoperative and procedural pain management, 2nd edition. *Paediatr Anaesth* 2012;22 Suppl 1(s1):1-79. doi: 10.1111/j.1460-9592.2012.03838.x [published Online First: 2012/08/01]
19. Royal College of Nursing, editor. *The recognition and assessment of acute pain in children: update of full guideline [Electronic version]*, 2009.
20. Taskforce DAS, Baron R, Binder A, et al. Evidence and consensus based guideline for the management of delirium, analgesia, and sedation in intensive care medicine. Revision 2015 (DAS-Guideline 2015) - short version. *Ger Med Sci* 2015;13:Doc19. doi: 10.3205/000223 [published Online First: 2015/11/27]

Confidential: For Review Only

21. Playfor S, Jenkins I, Boyles C, et al. Consensus guidelines on sedation and analgesia in critically ill children. *Intensive Care Med* 2006;32(8):1125-36. doi: 10.1007/s00134-006-0190-x [published Online First: 2006/05/16]
22. Sauder P, Andreoletti M, Cambonie G, et al. [Sedation and analgesia in intensive care (with the exception of new-born babies). French Society of Anesthesia and Resuscitation. French-speaking Resuscitation Society]. *Ann Fr Anesth Reanim* 2008;27(7-8):541-51. doi: 10.1016/j.annfar.2008.04.021 [published Online First: 2008/06/27]
23. Balice-Bourgois C, Zumstein-Shaha M, Vanoni F, et al. A Systematic Review of Clinical Practice Guidelines for Acute Procedural Pain on Neonates. *Clin J Pain* 2020;36(5):390-98. doi: 10.1097/AJP.0000000000000808 [published Online First: 2020/01/25]
24. Gamst-Jensen H, Vedel PN, Lindberg-Larsen VO, et al. Acute pain management in burn patients: appraisal and thematic analysis of four clinical guidelines. *Burns* 2014;40(8):1463-9. doi: 10.1016/j.burns.2014.08.020 [published Online First: 2014/10/04]
25. Lee GY, Yamada J, Kyololo O, et al. Pediatric clinical practice guidelines for acute procedural pain: a systematic review. *Pediatrics* 2014;133(3):500-15. doi: 10.1542/peds.2013-2744 [published Online First: 2014/02/04]
26. Eikermann M, Holzmann N, Siering U, et al. Tools for assessing the content of guidelines are needed to enable their effective use--a systematic comparison. *BMC Res Notes* 2014;7(1):853. doi: 10.1186/1756-0500-7-853 [published Online First: 2014/11/28]
27. McCurtin A, Boland P, Kavanagh M, et al. Do stroke clinical practice guideline recommendations for the intervention of thickened liquids for aspiration support evidence based decision making? A systematic review and narrative synthesis. *J Eval Clin Pract* 2020;26(6):1744-60. doi: 10.1111/jep.13372 [published Online First: 2020/02/23]
28. Cosgrove L, Bursztajn HJ, Erlich DR, et al. Conflicts of interest and the quality of recommendations in clinical guidelines. *J Eval Clin Pract* 2013;19(4):674-81. doi: 10.1111/jep.12016 [published Online First: 2013/06/05]
29. Liu Y, Zhang Y, Wang S, et al. Quality of pediatric clinical practice guidelines. *BMC Pediatr* 2021;21(1):223. doi: 10.1186/s12887-021-02693-1 [published Online First: 2021/05/09]
30. Siering U, Eikermann M, Hausner E, et al. Appraisal tools for clinical practice guidelines: a systematic review. *PLoS One* 2013;8(12):e82915. doi: 10.1371/journal.pone.0082915 [published Online First: 2013/12/19]
31. Brouwers MC, Kho ME, Browman GP, et al. AGREE II: advancing guideline development, reporting and evaluation in health care. *CMAJ* 2010;182(18):E839-42. doi: 10.1503/cmaj.090449 [published Online First: 2010/07/07]
32. Brouwers MC, Spithoff K, Kerkvliet K, et al. Development and Validation of a Tool to Assess the Quality of Clinical Practice Guideline Recommendations. *JAMA Netw Open* 2020;3(5):e205535. doi: 10.1001/jamanetworkopen.2020.5535 [published Online First: 2020/05/28]
33. Johnston A, Kelly SE, Hsieh SC, et al. Systematic reviews of clinical practice guidelines: a methodological guide. *J Clin Epidemiol* 2019;108:64-76. doi: 10.1016/j.jclinepi.2018.11.030 [published Online First: 2018/12/12]
34. Moher D, Shamseer L, Clarke M, et al. Preferred reporting items for systematic review and meta-analysis protocols (PRISMA-P) 2015 statement. *Syst Rev* 2015;4(1):1. doi: 10.1186/2046-4053-4-1 [published Online First: 2015/01/03]
35. McGowan J, Sampson M, Salzwedel DM, et al. PRESS Peer Review of Electronic Search Strategies: 2015 Guideline Statement. *J Clin Epidemiol* 2016;75:40-6. doi: 10.1016/j.jclinepi.2016.01.021 [published Online First: 2016/03/24]
36. Ouzzani M, Hammady H, Fedorowicz Z, et al. Rayyan-a web and mobile app for systematic reviews. *Syst Rev* 2016;5(1):210. doi: 10.1186/s13643-016-0384-4 [published Online First: 2016/12/07]

Confidential: For Review Only

37. Page MJ, Moher D, Bossuyt PM, et al. PRISMA 2020 explanation and elaboration: updated guidance and exemplars for reporting systematic reviews. *BMJ* 2021;372:n160. doi: 10.1136/bmj.n160 [published Online First: 2021/03/31]
38. The AGREE Next Steps Consortium. Appraisal of guidelines for research and evaluation II instrument - user manual. Ontario, CA: The Agree Research Trust 2010.
39. Koo TK, Li MY. A Guideline of Selecting and Reporting Intraclass Correlation Coefficients for Reliability Research. *J Chiropr Med* 2016;15(2):155-63. doi: 10.1016/j.jcm.2016.02.012 [published Online First: 2016/06/23]
40. Hoffmann-Esser W, Siering U, Neugebauer EAM, et al. Systematic review of current guideline appraisals performed with the Appraisal of Guidelines for Research & Evaluation II instrument-a third of AGREE II users apply a cut-off for guideline quality. *J Clin Epidemiol* 2018;95:120-27. doi: 10.1016/j.jclinepi.2017.12.009 [published Online First: 2017/12/31]

Confidential: For Review Only

SUPPLEMENTARY MATERIALS

Appendix A: PRISMA-P[1] (Preferred Reporting Items for Systematic review and Meta-Analysis Protocols) 2015 checklist: recommended items to address in a systematic review protocol

Section and topic	Item No	Checklist item	Page number (line)
**ADMINISTRATIVE INFORMATION**			
Title:			
Identification	1a	Identify the report as a protocol of a systematic review	6 (98-104) + title
Update	1b	If the protocol is for an update of a previous systematic review, identify as such	N/A
Registration	2	If registered, provide the name of the registry (such as PROSPERO) and registration number	Abstract + 6 (101-103)
Authors:			
Contact	3a	Provide name, institutional affiliation, e-mail address of all protocol authors; provide physical mailing address of corresponding author	1 (3-5 +table)
Contributions	3b	Describe contributions of protocol authors and identify the guarantor of the review	11 (227 – 230)
Amendments	4	If the protocol represents an amendment of a previously completed or published protocol, identify as such and list changes; otherwise, state plan for documenting important protocol amendments	6 (103-104)
Support:			
Sources	5a	Indicate sources of financial or other support for the review	11 (231-232)
Sponsor	5b	Provide name for the review funder and/or sponsor	N/A
Role of sponsor or funder	5c	Describe roles of funder(s), sponsor(s), and/or institution(s), if any, in developing the protocol	N/A
**INTRODUCTION**			
Rationale	6	Describe the rationale for the review in the context of what is already known	4-5 (36 - 88)
Objectives	7	Provide an explicit statement of the question(s) the review will address with reference to participants, interventions, comparators, and outcomes (PICO)	5 (88-96)
**METHODS**			
Eligibility criteria	8	Specify the study characteristics (such as PICO, study design, setting, time frame) and report characteristics (such as years considered, language, publication status) to be used as criteria for eligibility for the review	6-7 (106-115; Table 1: PICAR)
Information sources	9	Describe all intended information sources (such as electronic databases, contact with study authors, trial	7 (118 – 124)

Confidential: For Review Only

		registers or other grey literature sources) with planned dates of coverage	(Appendix B)
Search strategy	10	Present draft of search strategy to be used for at least one electronic database, including planned limits, such that it could be repeated	7-6 (126-130) (Appendix C)
Study records:			
Data management	11a	Describe the mechanism(s) that will be used to manage records and data throughout the review	8 (133 – 135)
Selection process	11b	State the process that will be used for selecting studies (such as two independent reviewers) through each phase of the review (that is, screening, eligibility and inclusion in meta-analysis)	8 (136 – 144)
Data collection process	11c	Describe planned method of extracting data from reports (such as piloting forms, done independently, in duplicate), any processes for obtaining and confirming data from investigators	8 (146 – 148)
Data items	12	List and define all variables for which data will be sought (such as PICO items, funding sources), any pre-planned data assumptions and simplifications	8-9 (150 – 159) (Appendix D)
Outcomes and prioritization	13	List and define all outcomes for which data will be sought, including prioritization of main and additional outcomes, with rationale	9 (175-177)
Risk of bias in individual studies	14	Describe anticipated methods for assessing risk of bias of individual studies, including whether this will be done at the outcome or study level, or both; state how this information will be used in data synthesis	9 (161-174)
Data synthesis	15a	Describe criteria under which study data will be quantitatively synthesized	N/A
	15b	If data are appropriate for quantitative synthesis, describe planned summary measures, methods of handling data and methods of combining data from studies, including any planned exploration of consistency (such as I^2, Kendall's τ)	N/A
	15c	Describe any proposed additional analyses (such as sensitivity or subgroup analyses, meta-regression)	N/A
	15d	If quantitative synthesis is not appropriate, describe the type of summary planned	9-10 (184 – 197)
Meta-bias(es)	16	Specify any planned assessment of meta-bias(es) (such as publication bias across studies, selective reporting within studies)	None
Confidence in cumulative evidence	17	Describe how the strength of the body of evidence will be assessed (such as GRADE)	10 (197 - 198)

N/A = not applicable

[1] Moher D, Shamseer L, Clarke M, et al. Preferred reporting items for systematic review and meta-analysis protocols (PRISMA-P) 2015 statement. *Systematic reviews* 2015;4(1):1.

Appendix B: Guideline repositories and Professional Societies/Organizations

	Country/Region	Website
**Guideline repositories**		
Australian Clinical Practice Guidelines Portal	Australia	https://www.clinicalguidelines.gov.au/portal
ECRI	US/UK	https://guidelines.ecri.org/
Guideline Central	US	https://www.guidelinecentral.com/summaries/#summary-view-specialty
Guidelines International Network (G-I-N)	Scotland	https://guidelines.ebmportal.com/
TRIP	UK	https://www.tripdatabase.com/
Up-to-date Society guideline links	Netherlands	http://www.uptodate.com/contents/search
**Professional societies/organizations**		
CISMeF Bonnes pratiques	France	https://doccismef.chu-rouen.fr/dc/#env=bp
European Society of Intensive Care Medicine (ESICM)	Europe	https://www.esicm.org/
European Society of Paediatric Neonatal Intensive Care (ESPNIC)	Europe	https://espnic-online.org
Infobanque Guides de Pratiques Clinique (GPC)	Canada	https://jouleamc.ca/cpg/homepage
National Institute for Health and Care Excellence (NICE)	UK	www.nice.org.uk
Registered Nurses' Association of Ontario (RNAO)	Canada	https://rnao.ca/bpg

Confidential: For Review Only

Royal College of Nursing	UK	https://www.rcn.org.uk/
Scottish Intercollegiate Guidelines Network (SIGN)	Scotland	http://sign.ac.uk/index.html
Société de Réanimation de Langue Française (SRLF)	France	https://srlf.org
Société Française d'Anesthésie et de Réanimation (SFAR)	France	https://sfar.org
Société Suisse de Pédiatrie	Switzerland	https://www.paediatrieschweiz.ch/fr/
Société Suisse de Médecine Intensive (SSMI)	Switzerland	https://www.sgi-ssmi.ch/fr
Society of Critical Care Medicine (SCCM)	USA	https://www.sccm.org/Home
World Federation of Pediatric Intensive and Critical Care Societies (WFPICCS)	Worldwide	httsp://wfpiccs.org

Confidential: For Review Only

Appendix C: Preliminary search strategy for Embase.org

('pain'/de OR 'analgesia'/exp OR 'conscious sedation'/de OR 'hypnotic sedative agent'/de OR 'delirium'/de OR 'withdrawal syndrome'/de OR (pain OR discomfort OR analgesia OR sedation OR sedative* OR delirium OR delirious* OR withdrawal*):ti,kw) AND ('practice guideline'/de OR 'consensus development'/de OR (guideline* OR cpg* OR guidance OR ((position OR policy) NEAR/3 (paper* OR development)) OR ((practice OR clinical) NEAR/3 development) OR (practice NEAR/2 guide$) OR recommendation* OR consensus OR standards OR statement*):ti,kw) NOT ('adult'/exp NOT 'juvenile'/exp) AND ([english]/lim OR [french]/lim) AND [2010-3000]/py NOT ('conference abstract'/it OR 'conference review'/it OR 'editorial'/it OR 'letter'/it) NOT ([animals]/lim NOT [humans]/lim)

7283 references on 27 July 2021

Confidential: For Review Only

Appendix D: Figures of excel spreadsheets for data collection

Figure 1. General Information

Title	First Author	Publication year	Language	Developing / publishing organisation and / or authors	Country / region of publication	How described by the authors (e.g. guideline / consensus statement / position stateme	Version	Topic addressed (pain) [insert 1]	Topic addressed (sedation) [insert 1]	Topic addressed (delirium) [insert 1]	Topic addressed (withdrawal) [insert 1]	Target population (A+adult, C=child, NC=non-communicative)[insert all that apply)	Target setting (G=general; S=specific) [specify e.g. G (acute care) S (PICU)	LoE rating system	GoE rating system

Figure 2. AGREE II

	CPG#1 [Name]		CPG#2 [Name]		CPG
**Domain 1: Scope and purpose**	Reviewer 1	Reviewer2	Reviewer 1	Reviewer2	Review
1. The overall objective(s) of the guideline is (are) specifically described					
2. The health question(s) covered by the guideline is (are) specifically described					
3. The population (patients, public, etc.) to whom the guideline is meant to apply is specifically described					
**Domain 2: Stakeholder involvement**					
4. The guideline development group includes individuals from all relevant professional groups					
5. The views and preferences of the target population (patients, public, etc.) have been sought					
6. The target users of the guideline are clearly defined					
**Domain 3: Rigour of development**					
7. Systematic methods were used to search for evidence					
8. The criteria for selecting the evidence are clearly described					
9. The strengths and limitations of the body of evidence are clearly described					
10. The methods for formulating the recommendations are clearly described					
11. The health benefits, side effects, and risks have been considered in formulating the recommendations					
12. There is an explicit link between the recommendations and the supporting evidence					
13. The guideline has been externally reviewed by experts prior to its publication					
14. A procedure for updating the guideline is provided					
**Domain 4: Clarity of presentation**					
15. The recommendations are specific and unambiguous					
16. The different options for management of the condition or health issue are clearly presented					
17. Key recommendations are easily identifiable					
**Domain 5: Applicability**					
18. The guideline describes facilitators and barriers to its application					
19. The guideline provides advice and/or tools on how the recommendations can be put into practice					
20. The potential resource implications of applying the recommendations have been considered					
21. The guideline presents monitoring and/or auditing criteria					
**Domain 6: Editorial independence**					
22. The views of the funding body have not influenced the content of the guideline					
23. Competing interests of guideline development group members have been recorded and addressed					
**Overall guideline assessment**					
1. Rate the overall quality of this guideline					

Figure 3. AGREE-REX

Guideline Name	CPG#1 [Name]		CPG#2 [Name]		CPG#3 [Na	
	Reviewer 1	Reviewer2	Reviewer 1	Reviewer2	Reviewer 1	Rev
**Domain 1: Clinical Applicability**						
1. Evidence						
2. Applicability to Target Users						
3. Applicability to Patients/Populations						
**Domain 2: Values and Preferences**						
4. Values and Preferences of Target Users						
5. Values and Preferences of Patients/Populations						
6. Values and Preferences of Policy/Decision-Makers						
7. Values and Preferences of Guideline Developers						
**Domain 3: Implementability**						
8. Purpose						
9. Local Application and Adoption						
1. I would recommend these guideline recommendations for use in the appropriate context.						
2. I would recommend these guideline recommendations for use in my context (optional).						

Figure 4. Recommendations and evidence (one table per concept)

Guideline Title	Recommendation	Grade of Recommendation	Evidence cited for recommendation (Author, year)	Recommendation category specified within guideline

BMJ Paediatrics Open

Quality of clinical practice guidelines and recommendations for the management of pain, sedation, delirium, and iatrogenic withdrawal in pediatric intensive care: A systematic review protocol

Journal:	*BMJ Paediatrics Open*
Manuscript ID	bmjpo-2021-001293.R1
Article Type:	Protocol
Date Submitted by the Author:	29-Nov-2021
Complete List of Authors:	MacDonald, Ibo; University of Lausanne, University Institute of Higher Education and Research in Healthcare<br>Perez, Marie-Hélène; Lausanne University Hospital Department of Woman Mother and Child<br>Trombert, Alexia; Lausanne University Hospital, Medical Library<br>Amiet, Vivianne; Lausanne University Hospital Department of Woman Mother and Child<br>Ramelet, Anne-Sylvie; University of Lausanne Faculty of Biology and Medicine, University Institute of Higher Education and Research in Healthcare
Keywords:	Pain

SCHOLARONE™
Manuscripts

I, the Submitting Author has the right to grant and does grant on behalf of all authors of the Work (as defined in the below author licence), an exclusive licence and/or a non-exclusive licence for contributions from authors who are: i) UK Crown employees; ii) where BMJ has agreed a CC-BY licence shall apply, and/or iii) in accordance with the terms applicable for US Federal Government officers or employees acting as part of their official duties; on a worldwide, perpetual, irrevocable, royalty-free basis to BMJ Publishing Group Ltd ("BMJ") its licensees and where the relevant Journal is co-owned by BMJ to the co-owners of the Journal, to publish the Work in this journal and any other BMJ products and to exploit all rights, as set out in our licence.

The Submitting Author accepts and understands that any supply made under these terms is made by BMJ to the Submitting Author unless you are acting as an employee on behalf of your employer or a postgraduate student of an affiliated institution which is paying any applicable article publishing charge ("APC") for Open Access articles. Where the Submitting Author wishes to make the Work available on an Open Access basis (and intends to pay the relevant APC), the terms of reuse of such Open Access shall be governed by a Creative Commons licence – details of these licences and which Creative Commons licence will apply to this Work are set out in our licence referred to above.

Other than as permitted in any relevant BMJ Author's Self Archiving Policies, I confirm this Work has not been accepted for publication elsewhere, is not being considered for publication elsewhere and does not duplicate material already published. I confirm all authors consent to publication of this Work and authorise the granting of this licence.

Confidential: For Review Only

Quality of clinical practice guidelines and recommendations for the management of pain, sedation, delirium, and iatrogenic withdrawal in pediatric intensive care: A systematic review protocol

Ibo MacDonald[1], Marie-Hélène Perez[2], Vivianne Amiet[2], Alexia Trombert[3], Anne-Sylvie Ramelet[1,2]*

Full name	Institution, Department	Postal address		e-mail	ORCID ID
*Corresponding author					
Anne-Sylvie Ramelet	University Institute of Higher Education and Research in Healthcare, Faculty of Biology and Medicine, University of Lausanne and Department Woman-Mother-Child, Lausanne University Hospital	Route de la Corniche 10, Lausanne, Switzerland, CH-1010		Anne-sylvie.ramelet@chuv.ch	0000-0001-8809-2920
Co-authors	Institution, Department	City	Country	e-mail	
Ibo MacDonald	University Institute of Higher Education and Research in Healthcare, Faculty of Biology and Medicine, University of Lausanne	Lausanne	Switzerland	Ibo.macdonald@chuv.ch	0000-0001-9490-8850
Marie-Hélène Perez	Lausanne University Hospital, Department Woman-Mother-Child and University of Lausanne			Marie-helene.perez@chuv.ch	0000-0001-6173-3396
Vivianne Amiet	Lausanne University Hospital, Department Woman-Mother-Child and University of Lausanne			Vivianne.Amiet@chuv.ch	0000-0002-6665-5291
Alexia Trombert	Lausanne University Hospital and University of Lausanne, Medical Library			Alexia.Trombert@chuv.ch	0000-0002-4833-5219

SUMMARY
Word count: 2747/2500
Abstract: 295/300
Tables/figures: 1/8
References: 43/40

Confidential: For Review Only

ABSTRACT (295/300)

Introduction: Inadequate management of pain and sedation in critically ill children can cause unnecessary suffering and agitation, but also delirium and iatrogenic withdrawal. It is therefore important to address these four interrelated conditions together. Some clinical practice guidelines (CPGs) are available for the management of pain and sedation, and a few for delirium and iatrogenic withdrawal in the PICU; none address the four conditions altogether. Critical appraisal of the quality of CPGs is necessary for their recommendations to be adopted into clinical practice. The aim of this systematic review is to identify and appraise the quality of CPGs and recommendations for management of either pain, sedation, delirium and iatrogenic withdrawal.

Methods and analysis: Researchers will conduct a systematic review in electronic databases (Medline ALL (Ovid), Embase.com, CINAHL with Full Text (EBSCO), JBI EBP Database (Ovid)), guideline repositories and websites of professional societies to identify CPGs published from 2010 to date. They will then combine index and free terms describing CPGs with pain, sedation, delirium and withdrawal. The researchers will include CPGs if they can be applied in the pediatric intensive care population (newborns to 18 years old) and include recommendation(s) for assessment of at least one of the four conditions. Two independent reviewers will screen for eligibility, complete data extraction and quality assessments using the Appraisal of Guidelines for Research and Evaluation (AGREE) II and the AGREE Recommendation Excellence (AGREE-REX) instruments. Researchers will report characteristics, content and recommendations from CPGs in tabulated forms.

Ethics and dissemination: Ethical approval is not required for this systematic review. Results will be published in a peer-reviewed journal.

Trial registration: This protocol is registered in the international database of prospectively registered systematic reviews (PROSPERO CRD42021274364).

Key words: AGREE II, children, clinical practice guideline, delirium, pain, sedation, systematic review, iatrogenic withdrawal

Confidential: For Review Only

34 **SUMMARIZING BOX**

**What is known about the subject** (2/3 statements)<br>• Optimal analgesia and sedation management is challenging in the pediatric intensive care unit. (13/25 words)<br>• Pain, sedation, delirium and iatrogenic withdrawal are interrelated conditions that need to be assessed as a whole to prevent negative outcomes. (21/25 words)
**What this study hopes to add** (2/3 statements)<br>• Researchers will compare the quality of clinical practice guidelines across all four interrelated conditions of pain, sedation, delirium and iatrogenic withdrawal. (21/25 words)<br>• Researchers will compare recommendations by describing the certainty and applicability of the base of evidence for clinical practice guidelines. (19/25 words)

35

Confidential: For Review Only

INTRODUCTION

Critically ill children in the pediatric intensive care unit (PICU), particularly those who are mechanically ventilated, require adequate identification and treatment of pain, sedation, delirium and iatrogenic withdrawal.[1] Healthcare professionals (HCPs) manage this with the use of analgesics and sedatives, often in combination, with 65% of children receiving both.[2] Optimal titration of analgesics and sedatives prevent pain, delirium and iatrogenic withdrawal,[3,4] as well as accidental extubation.[5] Over sedation and analgesia can result in prolonged mechanical ventilation, increased PICU length of stay, morbidity and mortality.[1] In the PICU setting 40-65% of children are unable to self-report due to mechanical ventilation and young age (under the age of four).[6,7] Given the large proportion of non-communicative children and the need for quality care, it is crucial that HCPs use multiple measurement instruments to assess pain, sedation, delirium and iatrogenic withdrawal.

HCPs have access to several measurement instruments for their use for assessing pain and sedation,[8] and more recently, for delirium and iatrogenic withdrawal.[9] When used, they help to individualize treatment. However, in a recent international survey study, researchers found wide variation in their application into practice.[10] In fact, some researchers have shown that HCPs struggle to select the right measurement instrument for these four conditions.[11,12] This may be due to the overlap among similar behavioural cue items across measurement instruments across these four conditions, or the plethora of measurement instruments available.[13,14] Pain and sedation, and delirium and withdrawal, are concomitant pairs. This is demonstrated by the development and use of measurement instruments for these pairs (e.g. COMFORT behaviour scale[15] and SOS-PD[16]). While on one hand there is a need for measurement instruments for better identification of these conditions separately, their separation can cause mismanagement. Thus assessment is the cornerstone for planning appropriate multi-modal interventions.[17] Overlaps exist between multi-modal interventions and the four conditions. Consequently, there has been a call from researchers to avoid looking at these conditions in siloes and to incorporate them together into standardized care practices,[13,14] either in clinical practice guidelines (CPGs) or as protocols or algorithms to improve assessment and management.

CPGs are created by synthesizing research to help bridge the evidence-to-practice gap. The purported benefits of CPG implementation are the standardization of care practices; improvements in patient safety; and patient outcomes.[18] There are few CPGs for

Confidential: For Review Only

management of these four conditions in the PICU, with only two for pain,[19,20] and one for pain, sedation and delirium.[21] Furthermore, some guidance documents exist, including practice/position/consensus statements/recommendations (hereafter, referred to as CPGs).[13,22,23] Although there is one position statement for the assessment of pain, sedation, delirium and iatrogenic withdrawal for pediatric critical care patients,[13] it does not include recommendations for management. Previous systematic reviews of CPGs for pain in pediatrics exist. They target neonates,[24] or burn patients,[25] or focus on procedural[24,26] or acute pain.[25] Systematic reviews of CPGs concerning best practices for children in the PICU are lacking. To date, none have been conducted on either of the four conditions, nor have they been examined together. Recently, scholars have criticized systematic reviews of CPGs and their lack of quality appraisal of recommendations.[27] Several systematic reviews of CPG recommendations have demonstrated evidence that weakly supports the recommendations.[28,29] Researchers, in a recent systematic review of CPGs for pediatric populations, that filtered publications between 2017 and 2019, found that 75% of the 216 CPGs were evidence-based.[30] It is important that researchers establish methodological quality of CPGs and the evidence base of recommendations to promote evidence-informed interventions.

Quality CPGs and their contained recommendations should reflect the most current evidence. Although, numerous appraisal instruments exist for assessing the quality of CPGs,[31] internationally, the appraisal of guidelines for research and evaluation (AGREE) II has emerged as the most widely used appraisal instrument.[32] The AGREE Enterprise recently developed the AGREE recommendation of excellence (AGREE-REX) instrument for the quality appraisal of recommendations.[33] Thus, researchers should use these two instruments jointly to assess CPG quality and to ensure that the evidence supporting recommendations in CPGs is reliable and trustworthy. This is essential so that CPGs remain sources of information that clinicians use to improve their practice and care of patients. The identification, appraisal and comparison of quality of CPGs and their recommendations is a valuable first step in informing efforts to incorporate these four overlapping conditions together in a standardized way to optimize care in the PICU. In this systematic review, researchers aim to appraise the quality of CPGs and recommendations for the management of pain, sedation, delirium and iatrogenic withdrawal in the PICU. Their specific objectives are:

1. To identify published CPGs for the management of pain, sedation, delirium and iatrogenic withdrawal;
2. To appraise the quality of selected CPGs;

Confidential: For Review Only

3. To appraise the quality of recommendations included in CPGs; and
4. To summarize the convergence of recommendations and the overall robustness of recommendation in CPGs.

METHODS AND ANALYSIS

Researchers of this study protocol used the methodological guide for conducting systematic reviews of CPGs to guide the development of each stage.[34] They reported according to the preferred reporting items for systematic review and meta-analysis protocols (PRISMA-P) (online supplemental table 1A).[35] The researchers registered the study protocol in the international database of prospectively registered systematic reviews (PROSPERO ID CRD42021274364, available from: https://www.crd.york.ac.uk/prospero/display_record.php?ID=CRD42021274364). In the event of amendment to this protocol, the PROSPERO register will be updated.

Inclusion/exclusion criteria for study selection

To guide CPG selection, the population, intervention, comparators, attributes and recommendations (PICAR) framework was used (see Table 1).[34] For the purpose of this review, CPGs must have included recommendations developed from available evidence, including expert opinion.[18] CPGs and guidance statements will be considered. CPGs that include pediatric populations will be included in this review if they: i) are endorsed by a society, ii) include a recommendation for assessment of any of the four conditions, and iii) are the most current version. Publication year will be limited to 2010 to present for two reasons. Firstly, this timeframe corresponds with a paradigmatic shift in intensive care unit sedation practice.[36] Secondly, the first consensus guideline for critically ill children was published in 2006.[22] If updated within 5 years, as recommended,[37] it would be captured within the search strategy. This review will include broad CPGs for the assessment and management of any of the four conditions, including postoperative pain. However, CPGs will be excluded if focused on specific patient groups (e.g. cardiac). CPGs on diagnostic procedures (e.g. endoscopy) or procedures of limited temporal duration (e.g. venipuncture) will be excluded. Procedures that require prolonged use in the PICU setting (e.g. respiratory support), will be included.

Table 1: PICAR statement: inclusion and exclusion criteria

**P**opulation, clinical indication(s), and condition(s)	**Study population**:<br>- Include: Children (newborn (>38 weeks gestations) to 18 years of age)

Confidential: For Review Only

	- Exclude: premature infants and adults<br>**Clinical indications**:<br>- Include: management of either pain (including postoperative, persistent and prolonged pain), sedation, delirium or iatrogenic withdrawal<br>- Exclude: Management specific to medications, chronic and procedural pain of short duration, procedural sedation provided in other care settings (e.g. dentistry, radiology, endoscopy) or for short duration<br>**Conditions**:<br>- Include: children in intensive care
**I**nterventions	Any intervention focusing on the on-going management of either pain, sedation, delirium or iatrogenic withdrawal
**C**omparator(s), comparison(s), and (key) content	**Comparator/comparison:** Any<br>**Key content**:<br>- If a broad population is included, the CPG must have separate recommendations for children<br>- The CPG can be implemented in the intensive care setting but does not need to be specifically developed for intensive care<br>- The CPG must include recommendations on assessment for either pain, sedation, delirium or iatrogenic withdrawal
**A**ttributes of eligible CPGs	**Language:** No restrictions<br>**Year of publication**: 2010 onward<br>**Setting**:<br>- Include: Applicable to pediatric intensive care, can be broad/general<br>- Exclude: CPGs developed specifically for other settings: neonatal intensive care units, emergency department, pre-operative/operating room<br>**Developing/publishing organization:**<br>- Include: CPGs issued or endorsed by international, national or regional societies/professional organizations, or governments from developed countries<br>- Exclude: CPGs that were developed by an individual organization (e.g. hospital) or unit within an organization<br>**Version**: Latest/newest version (preceding versions will be excluded)<br>**Type**: CPGs, consensus statements, practice/position recommendations/alerts/statements<br>**Quality score**: The AGREE II will be used to assess quality but will not be used as a criterion to determine eligibility for inclusion in this systematic review
**R**ecommendations characteristics and "other" considerations	**Recommendations:** CPGs must have at least one specific recommendation for assessment for either pain, sedation, delirium or iatrogenic withdrawal (either explicitly highlighted as a recommendation (primary) or noted in the body of the text (secondary - not explicitly identified as a recommendation))

Search methods

Confidential: For Review Only

Informational sources

The search will be conducted in:

1) Four electronic databases: Medline ALL (Ovid), Embase.com CINAHL with Full Text (EBSCO), and Joanna Briggs Institute (JBI) EBP Database.
2) Eight guideline repositories.
3) Fourteen professional societies/organizations (online supplemental table 1B contains a list of guideline repositories and professional societies/organizations to be searched).

Search strategy

The search strategy will be developed with the assistance of a health services librarian. Index and free terms describing CPGs and pain, sedation, delirium, withdrawal will be combined to create an advanced search strategy that will be translated for all databases and sources of information. The final search strategy for Embase.com is provided in the online supplemental table 1C. The search strategy will be peer reviewed by another librarian using the PRESS checklist.[38] During full-text screening, if a CPG is mentioned, it will be retrieved for review.

Guideline selection

The search results will be imported into Endnote 20 reference manager (Clarivate Analytics, USA) for duplicate removal. The remaining citations will be uploaded to Rayyan QCRI (Qatar Computing Research Institute, Doha, Qatar) to manage the screening process.[39]

Titles and abstracts of all citations will be screened by two independent reviewers to determine those for full-text review. These will be retrieved and assessed against the inclusion and exclusion criteria. Reasons for exclusion will be recorded. Any disagreements will be resolved through discussion and consensus or by a third reviewer.

Supporting documents (e.g. evidence tables, conflict of interest declarations), where available, will be retrieved by the review team from the endorsing organization's website to ensure all relevant documents will be available for quality appraisal for included CPGs.

The PRISMA flow diagram will be used to show the selection process and summarise the inclusion and exclusion details.[40]

Data extraction

Confidential: For Review Only

Information from each included CPGs will be extracted by two independent reviewers. The review team developed an Excel spreadsheet for data extraction (online supplemental figures 1D to 4D) that will be piloted and revised during the data extraction phase. The following key areas will be extracted:

a) General information: title, first author, year of publication, language, developing organization, country, type of CPG, condition addressed (pain, sedation, delirium, iatrogenic withdrawal), target population, target setting, level of evidence (LoE) rating system, grade of evidence (GoE) rating system.
b) Quality of included CPGs using the AGREE II instrument (details below).[32]
c) Quality of recommendations from medium quality and higher CPGs using the AGREE-REX instrument (details below).[33]
d) Recommendations (one worksheet per condition, and each line will represent one recommendation): recommendation, grade of recommendation, classification of evidence, list of supporting citation(s), categorization of recommendation as per CPG.

Quality appraisal of CPGs and recommendations

Quality of CPGs. Each included CPG will be independently appraised by at least two reviewers using the AGREE II instrument.[32] The AGREE II is a validated and reliable appraisal instrument for assessing the quality of CPGs.[32] It contains 23 items across six domains: 1) scope and purpose; 2) stakeholder involvement; 3) rigour of development; 4) clarity of presentation; 5) applicability; and 6) editorial independence. Each item will be appraised against a 7-point Likert scale ranging from 1 (strongly disagree) to 7 (strongly agree). In addition, there are two global rating scores: 1) overall quality of the CPG, and 2) whether the guideline would be recommended for use.

Quality of CPGs recommendations. The AGREE–REX will be used for assessing the quality of recommendations.[33] It is a recently developed, valid and reliable appraisal instrument containing nine items across three domains: 1) clinical applicability; 2) values and preferences; and 3) implementability.[33] Each item will be appraised by at least three reviewers using the same seven-point Likert scale, as in the AGREE II. It includes a global rating score of the overall quality of the CPGs recommendations.

To score the AGREE II each item's score across reviewers will be summed and converted to a percentage of the maximum possible score for each domain.[32] For the

Confidential: For Review Only

AGREE-REX, the consensus score approach will be used, whereby the review team will meet to agree on AGREE-REX item scores [41]

To ensure standardization of appraisal, the training tools available for the AGREE II on the website (www.agreetrust.com) will be used to train each reviewer. For the AGREE-REX, a training video will be created by one research team member (IMD) using the sample guideline from the AGREE II training. The video will be used to train each member of the review team. One included CPG will then be selected by the entire review team, and a consensus meeting will be held to ensure familiarity with the tools. The AGREE II inter-rater agreement will be calculated using intra-class correlation coefficients (ICCs) with a two-way random effects model for each domain. The levels of ICC agreement will be classified as poor (<0.50), moderate (0.50–0.75), good (0.75–0.9), and excellent (>0.9).[42]

Data synthesis

Quality of CPGs and recommendations

The AGREE Enterprise has no established quality threshold, instead review teams must establish their own prior to appraisal.[43] As recommended in a recent systematic review of AGREE II thresholds for determining CPG quality, we will use the 3-step system where high quality are scores ≥ 60%, medium quality are scores between ≥30% - 60%, and low quality are scores < 30% across all domains.[44] For determining when the AGREE-REX will be applied to assessing recommendations the same a priori establishment of a threshold is recommended in the AGREE-REX user manual [43]. For this review, the AGREE-REX will be used only with CPGs that meet at least the medium level threshold (e.g. ≥ 30%) for methodological development using the AGREE II. Clusters of recommendations on single topics (e.g. assessment) will then be appraised in these CPGs. This decision was made because this is the first review of its kind, and the quality of recommendations between CPGs is uncertain.

The results of the AGREE II and AGREE-REX scores will be presented in a table. The quality of each domain will be presented as a heat map based on the threshold cut-offs for quality as described above.

Synthesis of recommendations and their level of evidence

For medium and high methodological quality CPGs (based on the AGREE II), all recommendations and their related evidence will be extracted per condition. Once extracted,

Confidential: For Review Only

each recommendation will be categorized based on the type of care intervention, including: a) prevention, b) assessment, and c) management. Management interventions will be further subdivided into pharmacological and non-pharmacological. Summary tables will be created to highlight the consistency of all recommendations for each condition. The LoE associated with recommendations within each CPG will be reported but not standardized across CPGs.

ETHICS AND DISSEMINATION

Ethical approval is not required for this systematic review. To disseminate the results the study will be published in a peer-reviewed journal.

PATIENT AND PUBLIC INVOLVEMENT

There was no patient or public involvement in the development of the systematic review protocol. The Swiss Society of Intensive Care Medicines' Pain, Agitation, Delirium, Immobility and Sleep working group (Swiss PADIS-WG) will be involved in data synthesis as clinical experts.

DISCUSSION

This systematic review of CPGs will generate a succinct and comprehensive summary of the best available evidence for the management of pain, sedation, delirium and iatrogenic withdrawal. This will be a valuable first step towards standardizing the management of pain, sedation, delirium and iatrogenic withdrawal in the PICU. Based on quality appraisal, the results will establish which CPGs can be recommended for use in clinical practice. It will also provide accessible summaries of the best evidence for each recommendation and type of care intervention for the four conditions to support implementation into practice. These results can be used as the basis for the development of a combined CPG for these four conditions specific for the PICU. This will in turn help to identify recommendation gaps, inconsistencies and lack of high quality evidence.

Research on pain, sedation, delirium and iatrogenic withdrawal practices across an international sample of 161 PICUs continues to demonstrate variation.[9] Although HCPs use measurement instruments to identify patient changes based on behavioural cues, HCPs may find it challenging to interpret and use scores and determine which multi-modal interventions to

Confidential: For Review Only

use. Using measurement instruments is the first step towards goal-directed care, and this review will give HCPs strategies to inform clinical practice.

The strengths of this systematic review are the comprehensive search for CPGs on the four interrelated conditions, which has not been previously conducted, as well as the evaluation of the quality of CPGs and the base of evidence for included recommendations.

A limitation of this systematic review will be ensuring the review team is sufficiently trained in using the AGREE-REX, as this is a novel tool. Currently, no training resources exist and guidance on its use during the systematic review process is lacking. This might lead to divergent scores. To mitigate this limitation, a member of the review team will develop a training video, and the researchers will use a consensus process. It is likely that the heterogeneity of research conducted in the PICU setting will lead to low-quality scores for recommendations.

CONCLUSION

Management of pain and sedation is a balancing act for HCPs in order to provide optimal comfort and avoid delirium and iatrogenic withdrawal for their pediatric patients. Recommendations for managing these four interrelated conditions are mixed. This systematic review will use rigorous methods to assess the quality and content of CPGs and included recommendations for the management of these four conditions. It will add to the current body of knowledge with the intention to optimise care and outcomes for critically ill pediatric patients in the PICU.

AUTHOR CONTRIBUTIONS.

The conception and design of this systematic review protocol was completed by three authors (IMD, ASR and MHP). The search strategy was developed by IMD and AT with feedback from the review team (ASR, MHP and VA). IMD prepared the first draft of the manuscript with all authors providing feedback towards the final manuscript.

FUNDING STATEMENT.

This research received no specific grant from any funding agency in the public, commercial or not-for-profit sectors.

COMPETING INTERESTES.

None declared

Confidential: For Review Only

Confidential: For Review Only

REFERENCES

1. Egbuta C, Mason KP. Current State of Analgesia and Sedation in the Pediatric Intensive Care Unit. *J Clin Med* 2021;10(9):1847. doi: 10.3390/jcm10091847 [published Online First: 2021/05/01]
2. Patel AK, Trujillo-Rivera E, Faruqe F, et al. Sedation, Analgesia, and Neuromuscular Blockade: An Assessment of Practices From 2009 to 2016 in a National Sample of 66,443 Pediatric Patients Cared for in the ICU. *Pediatr Crit Care Med* 2020;21(9):e599-e609. doi: 10.1097/PCC.0000000000002351 [published Online First: 2020/03/21]
3. Best KM, Boullata JI, Curley MA. Risk factors associated with iatrogenic opioid and benzodiazepine withdrawal in critically ill pediatric patients: a systematic review and conceptual model. *Pediatr Crit Care Med* 2015;16(2):175-83. doi: 10.1097/PCC.0000000000000306 [published Online First: 2015/01/07]
4. Mody K, Kaur S, Mauer EA, et al. Benzodiazepines and Development of Delirium in Critically Ill Children: Estimating the Causal Effect. *Crit Care Med* 2018;46(9):1486-91. doi: 10.1097/CCM.0000000000003194 [published Online First: 2018/05/05]
5. Lucas da Silva PS, de Carvalho WB. Unplanned extubation in pediatric critically ill patients: a systematic review and best practice recommendations. *Pediatr Crit Care Med* 2010;11(2):287-94. doi: 10.1097/PCC.0b013e3181b80951 [published Online First: 2009/10/02]
6. Ibiebele I, Algert CS, Bowen JR, et al. Pediatric admissions that include intensive care: a population-based study. *BMC Health Serv Res* 2018;18(1):264. doi: 10.1186/s12913-018-3041-x [published Online First: 2018/04/11]
7. Laures E, LaFond C, Hanrahan K, et al. Pain Assessment Practices in the Pediatric Intensive Care Unit. *J Pediatr Nurs* 2019;48:55-62. doi: 10.1016/j.pedn.2019.07.005 [published Online First: 2019/07/22]
8. Giordano V, Edobor J, Deindl P, et al. Pain and Sedation Scales for Neonatal and Pediatric Patients in a Preverbal Stage of Development: A Systematic Review. *JAMA Pediatr* 2019;173(12):1186-97. doi: 10.1001/jamapediatrics.2019.3351 [published Online First: 2019/10/15]
9. Ista E, Redivo J, Kananur P, et al. Assessing Pain, Both Spontaneous Awakening and Breathing Trials, Choice of Sedation, Delirium Monitoring/Management, Early Exercise/Mobility, and Family Engagement/Empowerment Bundle Practices for Critically Ill Children: An International Survey of 161 PICUs in 18 Countries. *Critical Care Medicine* 2021 doi: 10.1097/CCM.0000000000005168 [published Online First: July 13, 2021]
10. Madden K, Burns MM, Tasker RC. Differentiating Delirium From Sedative/Hypnotic-Related Iatrogenic Withdrawal Syndrome: Lack of Specificity in Pediatric Critical Care Assessment Tools. *Pediatr Crit Care Med* 2017;18(6):580-88. doi: 10.1097/PCC.0000000000001153 [published Online First: 2017/04/22]
11. Flaigle MC, Ascenzi J, Kudchadkar SR. Identifying Barriers to Delirium Screening and Prevention in the Pediatric ICU: Evaluation of PICU Staff Knowledge. *J Pediatr Nurs* 2016;31(1):81-84. doi: https://doi.org/10.1016/j.pedn.2015.07.009
12. LaFond CM, Hanrahan KS, Pierce NL, et al. Pain in the pediatric intensive care unit: How and what are we doing? *American Journal of Critical Care* 2019;28(4):265-73. doi: 10.4037/ajcc2019836
13. Harris J, Ramelet AS, van Dijk M, et al. Clinical recommendations for pain, sedation, withdrawal and delirium assessment in critically ill infants and children: an ESPNIC position statement for healthcare professionals. *Intensive Care Med* 2016;42(6):972-86. doi: 10.1007/s00134-016-4344-1 [published Online First: 2016/04/17]
14. Ista E, van Dijk M. We can not compartmentalize our patients! Overlapping symptoms of iatrogenic withdrawal syndrome, pediatric delirium, and anticholinergic toxidrome. *Pediatric critical care medicine* 2017;18(6):603-04.doi: 10.1097/PCC.0000000000001163

Confidential: For Review Only

15. van Dijk M, de Boer JB, Koot HM, et al. The reliability and validity of the COMFORT scale as a postoperative pain instrument in 0 to 3-year-old infants. *Pain* 2000;84(2):367-77. doi: 10.1016/s0304-3959(99)00239-0
16. Ista E, Te Beest H, van Rosmalen J, et al. Sophia Observation withdrawal Symptoms-Paediatric Delirium scale: A tool for early screening of delirium in the PICU. *Australian critical care : official journal of the Confederation of Australian Critical Care Nurses* 2018;31(5):266-73. doi: 10.1016/j.aucc.2017.07.006 [published Online First: 2017/08/28]
17. Stonehouse D. Understanding the nursing process. *British Journal of Healthcare Assistants* 2017;11(8):388-91. doi: 10.12968/bjha.2017.11.8.388
18. Graham R. Clinical practice guidelines we can trust. Washington, DC: National Academies Press 2011.
19. Association of Paediatric Anaesthetists of Great B, Ireland. Good practice in postoperative and procedural pain management, 2nd edition. *Paediatr Anaesth* 2012;22 Suppl 1(s1):1-79. doi: 10.1111/j.1460-9592.2012.03838.x [published Online First: 2012/08/01]
20. Royal College of Nursing, editor. *The recognition and assessment of acute pain in children: update of full guideline [Electronic version]*, 2009.
21. Taskforce DAS, Baron R, Binder A, et al. Evidence and consensus based guideline for the management of delirium, analgesia, and sedation in intensive care medicine. Revision 2015 (DAS-Guideline 2015) - short version. *Ger Med Sci* 2015;13:Doc19. doi: 10.3205/000223 [published Online First: 2015/11/27]
22. Playfor S, Jenkins I, Boyles C, et al. Consensus guidelines on sedation and analgesia in critically ill children. *Intensive Care Med* 2006;32(8):1125-36. doi: 10.1007/s00134-006-0190-x [published Online First: 2006/05/16]
23. Sauder P, Andreoletti M, Cambonie G, et al. [Sedation and analgesia in intensive care (with the exception of new-born babies). French Society of Anesthesia and Resuscitation. French-speaking Resuscitation Society]. *Ann Fr Anesth Reanim* 2008;27(7-8):541-51. doi: 10.1016/j.annfar.2008.04.021 [published Online First: 2008/06/27]
24. Balice-Bourgois C, Zumstein-Shaha M, Vanoni F, et al. A Systematic Review of Clinical Practice Guidelines for Acute Procedural Pain on Neonates. *Clin J Pain* 2020;36(5):390-98. doi: 10.1097/AJP.0000000000000808 [published Online First: 2020/01/25]
25. Gamst-Jensen H, Vedel PN, Lindberg-Larsen VO, et al. Acute pain management in burn patients: appraisal and thematic analysis of four clinical guidelines. *Burns* 2014;40(8):1463-9. doi: 10.1016/j.burns.2014.08.020 [published Online First: 2014/10/04]
26. Lee GY, Yamada J, Kyololo O, et al. Pediatric clinical practice guidelines for acute procedural pain: a systematic review. *Pediatrics* 2014;133(3):500-15. doi: 10.1542/peds.2013-2744 [published Online First: 2014/02/04]
27. Eikermann M, Holzmann N, Siering U, et al. Tools for assessing the content of guidelines are needed to enable their effective use--a systematic comparison. *BMC Res Notes* 2014;7(1):853. doi: 10.1186/1756-0500-7-853 [published Online First: 2014/11/28]
28. McCurtin A, Boland P, Kavanagh M, et al. Do stroke clinical practice guideline recommendations for the intervention of thickened liquids for aspiration support evidence based decision making? A systematic review and narrative synthesis. *J Eval Clin Pract* 2020;26(6):1744-60. doi: 10.1111/jep.13372 [published Online First: 2020/02/23]
29. Cosgrove L, Bursztajn HJ, Erlich DR, et al. Conflicts of interest and the quality of recommendations in clinical guidelines. *J Eval Clin Pract* 2013;19(4):674-81. doi: 10.1111/jep.12016 [published Online First: 2013/06/05]
30. Liu Y, Zhang Y, Wang S, et al. Quality of pediatric clinical practice guidelines. *BMC Pediatr* 2021;21(1):223. doi: 10.1186/s12887-021-02693-1 [published Online First: 2021/05/09]

Confidential: For Review Only

31. Siering U, Eikermann M, Hausner E, et al. Appraisal tools for clinical practice guidelines: a systematic review. *PLoS One* 2013;8(12):e82915. doi: 10.1371/journal.pone.0082915 [published Online First: 2013/12/19]
32. Brouwers MC, Kho ME, Browman GP, et al. AGREE II: advancing guideline development, reporting and evaluation in health care. *CMAJ* 2010;182(18):E839-42. doi: 10.1503/cmaj.090449 [published Online First: 2010/07/07]
33. Brouwers MC, Spithoff K, Kerkvliet K, et al. Development and Validation of a Tool to Assess the Quality of Clinical Practice Guideline Recommendations. *JAMA Netw Open* 2020;3(5):e205535. doi: 10.1001/jamanetworkopen.2020.5535 [published Online First: 2020/05/28]
34. Johnston A, Kelly SE, Hsieh SC, et al. Systematic reviews of clinical practice guidelines: a methodological guide. *J Clin Epidemiol* 2019;108:64-76. doi: 10.1016/j.jclinepi.2018.11.030 [published Online First: 2018/12/12]
35. Moher D, Shamseer L, Clarke M, et al. Preferred reporting items for systematic review and meta-analysis protocols (PRISMA-P) 2015 statement. *Syst Rev* 2015;4(1):1. doi: 10.1186/2046-4053-4-1 [published Online First: 2015/01/03]
36. Devabhakthuni S, Armahizer MJ, Dasta JF, et al. Analgosedation: a paradigm shift in intensive care unit sedation practice. *Ann Pharmacother* 2012;46(4):530-40. doi: 10.1345/aph.1Q525 [published Online First: 2012/04/13]
37. Vernooij RW, Sanabria AJ, Sola I, et al. Guidance for updating clinical practice guidelines: a systematic review of methodological handbooks. *Implement Sci* 2014;9(1):3. doi: 10.1186/1748-5908-9-3 [published Online First: 2014/01/05]
38. McGowan J, Sampson M, Salzwedel DM, et al. PRESS Peer Review of Electronic Search Strategies: 2015 Guideline Statement. *J Clin Epidemiol* 2016;75:40-6. doi: 10.1016/j.jclinepi.2016.01.021 [published Online First: 2016/03/24]
39. Ouzzani M, Hammady H, Fedorowicz Z, et al. Rayyan-a web and mobile app for systematic reviews. *Syst Rev* 2016;5(1):210. doi: 10.1186/s13643-016-0384-4 [published Online First: 2016/12/07]
40. Page MJ, Moher D, Bossuyt PM, et al. PRISMA 2020 explanation and elaboration: updated guidance and exemplars for reporting systematic reviews. *BMJ* 2021;372:n160. doi: 10.1136/bmj.n160 [published Online First: 2021/03/31]
41. AGREE-REX Research Team. The Appraisal of Guidelines Research & Evaluation – Recommendation Excellence (AGREE-REX) Instruction Manual. 2019.
42. Koo TK, Li MY. A Guideline of Selecting and Reporting Intraclass Correlation Coefficients for Reliability Research. *J Chiropr Med* 2016;15(2):155-63. doi: 10.1016/j.jcm.2016.02.012 [published Online First: 2016/06/23]
43. The AGREE Next Steps Consortium. Appraisal of guidelines for research and evaluation II instrument - user manual. Ontario, CA: The Agree Research Trust 2010.
44. Hoffmann-Esser W, Siering U, Neugebauer EAM, et al. Systematic review of current guideline appraisals performed with the Appraisal of Guidelines for Research & Evaluation II instrument-a third of AGREE II users apply a cut-off for guideline quality. *J Clin Epidemiol* 2018;95:120-27. doi: 10.1016/j.jclinepi.2017.12.009 [published Online First: 2017/12/31]

Confidential: For Review Only

SUPPLEMENTARY MATERIALS

Table 1A: PRISMA-P[1] (Preferred Reporting Items for Systematic review and Meta-Analysis Protocols) 2015 checklist: recommended items to address in a systematic review protocol

Section and topic	Item No	Checklist item	Page number (line)
**ADMINISTRATIVE INFORMATION**			
Title:			
Identification	1a	Identify the report as a protocol of a systematic review	6 (105 - 112) + title
Update	1b	If the protocol is for an update of a previous systematic review, identify as such	N/A
Registration	2	If registered, provide the name of the registry (such as PROSPERO) and registration number	Abstract + 6 (108-112)
Authors:			
Contact	3a	Provide name, institutional affiliation, e-mail address of all protocol authors; provide physical mailing address of corresponding author	1 (3-5 +table)
Contributions	3b	Describe contributions of protocol authors and identify the guarantor of the review	12 (270 – 273)
Amendments	4	If the protocol represents an amendment of a previously completed or published protocol, identify as such and list changes; otherwise, state plan for documenting important protocol amendments	6 (111-112)
Support:			
Sources	5a	Indicate sources of financial or other support for the review	12 (275-276)
Sponsor	5b	Provide name for the review funder and/or sponsor	N/A
Role of sponsor or funder	5c	Describe roles of funder(s), sponsor(s), and/or institution(s), if any, in developing the protocol	N/A
**INTRODUCTION**			
Rationale	6	Describe the rationale for the review in the context of what is already known	4-5 (36 -95)
Objectives	7	Provide an explicit statement of the question(s) the review will address with reference to participants, interventions, comparators, and outcomes (PICO)	5-6 (95-103)
**METHODS**			
Eligibility criteria	8	Specify the study characteristics (such as PICO, study design, setting, time frame) and report characteristics (such as years considered, language, publication status) to be used as criteria for eligibility for the review	6-7 (113-128; Table 1: PICAR)
Information sources	9	Describe all intended information sources (such as electronic databases, contact with study authors, trial registers or other grey literature sources) with planned dates of coverage	8 (132 – 139) (supplement table 1B)

Confidential: For Review Only

Search strategy	10	Present draft of search strategy to be used for at least one electronic database, including planned limits, such that it could be repeated	8 (140-146) (supplement table 1C)
Study records:			
Data management	11a	Describe the mechanism(s) that will be used to manage records and data throughout the review	8 (148 – 150)
Selection process	11b	State the process that will be used for selecting studies (such as two independent reviewers) through each phase of the review (that is, screening, eligibility and inclusion in meta-analysis)	8 (150 – 157)
Data collection process	11c	Describe planned method of extracting data from reports (such as piloting forms, done independently, in duplicate), any processes for obtaining and confirming data from investigators	9 (161 – 164)
Data items	12	List and define all variables for which data will be sought (such as PICO items, funding sources), any pre-planned data assumptions and simplifications	9 (165 – 174) (supplement tables 1D – 4D)
Outcomes and prioritization	13	List and define all outcomes for which data will be sought, including prioritization of main and additional outcomes, with rationale	9 - 10 (190-193)
Risk of bias in individual studies	14	Describe anticipated methods for assessing risk of bias of individual studies, including whether this will be done at the outcome or study level, or both; state how this information will be used in data synthesis	9 (176-189)
Data synthesis	15a	Describe criteria under which study data will be quantitatively synthesized	N/A
	15b	If data are appropriate for quantitative synthesis, describe planned summary measures, methods of handling data and methods of combining data from studies, including any planned exploration of consistency (such as I^2, Kendall's τ)	N/A
	15c	Describe any proposed additional analyses (such as sensitivity or subgroup analyses, meta-regression)	N/A
	15d	If quantitative synthesis is not appropriate, describe the type of summary planned	10-11 (216 – 226)
Meta-bias(es)	16	Specify any planned assessment of meta-bias(es) (such as publication bias across studies, selective reporting within studies)	None
Confidence in cumulative evidence	17	Describe how the strength of the body of evidence will be assessed (such as GRADE)	11 (205 - 215)

N/A = not applicable

[1] Moher D, Shamseer L, Clarke M, et al. Preferred reporting items for systematic review and meta-analysis protocols (PRISMA-P) 2015 statement. *Systematic reviews* 2015;4(1):1.

Confidential: For Review Only

Table 1B: Guideline repositories and Professional Societies/Organizations

	Country/Region	Website
**Guideline repositories**		
Australian Clinical Practice Guidelines Portal	Australia	https://www.clinicalguidelines.gov.au/portal
CISMeF Bonnes pratiques	France	https://doccismef.chu-rouen.fr/dc/#env=bp
ECRI	USA/UK	https://guidelines.ecri.org/
Guideline Central	USA	https://www.guidelinecentral.com/summaries/#summary-view-specialty
Guidelines International Network (G-I-N)	Scotland	https://guidelines.ebmportal.com/
Infobanque Guides de Pratiques Clinique (GPC)	Canada	https://jouleamc.ca/cpg/homepage
TRIP	UK	https://www.tripdatabase.com/
Up-to-date Society guideline links	Netherlands	http://www.uptodate.com/contents/search
**Professional societies/organizations**		
American Association of Critical-care Nurses	USA	https://www.aacn.org/clinical-resources/view-all-issues?category=practice-alerts
BIGG International database of GRADE guidelines	Worldwide	https://sites.bvsalud.org/bigg/en/biblio/
European Society of Intensive Care Medicine (ESICM)	Europe	https://www.esicm.org/
European Society of Paediatric Neonatal Intensive Care (ESPNIC)	Europe	https://espnic-online.org

Confidential: For Review Only

National Institute for Health and Care Excellence (NICE)	UK	www.nice.org.uk
Registered Nurses' Association of Ontario (RNAO)	Canada	https://rnao.ca/bpg
Royal College of Nursing	UK	https://www.rcn.org.uk/
Scottish Intercollegiate Guidelines Network (SIGN)	Scotland	http://sign.ac.uk/index.html
Société de Réanimation de Langue Française (SRLF)	France	https://srlf.org
Société Française d'Anesthésie et de Réanimation (SFAR)	France	https://sfar.org
Société Suisse de Pédiatrie	Switzerland	https://www.paediatrieschweiz.ch/fr/
Société Suisse de Médecine Intensive (SSMI)	Switzerland	https://www.sgi-ssmi.ch/fr
Society of Critical Care Medicine (SCCM)	USA	https://www.sccm.org/Home
World Federation of Pediatric Intensive and Critical Care Societies (WFPICCS)	Worldwide	httsp://wfpiccs.org

Confidential: For Review Only

Table 1C: Final search strategy for Embase.com

('pain'/de OR 'postoperative pain'/de OR 'analgesia'/exp OR 'conscious sedation'/de OR 'hypnotic sedative agent'/de OR 'delirium'/de OR 'hyperactive delirium'/de OR 'hypoactive delirium'/de OR 'withdrawal syndrome'/de OR (pain OR discomfort OR analgesia OR sedation OR sedative* OR delirium OR delirious* OR withdrawal*):ti,kw) AND ('practice guideline'/de OR 'consensus development'/de OR ((expert* NEAR/3 opinion*) OR guideline* OR cpg* OR guidance OR ((position OR policy) NEAR/3 (paper* OR development)) OR ((practice OR clinical) NEAR/3 development) OR (practice NEAR/2 guide$) OR recommendation* OR consensus OR standards OR statement*):ti,kw) NOT ('adult'/exp NOT 'juvenile'/exp) AND [2010-3000]/py NOT ('conference abstract'/it OR 'conference review'/it) NOT ([animals]/lim NOT [humans]/lim)<br><br>9849 references (17 November 2021)

Confidential: For Review Only

Figures 1D – 4D: Figures of excel spreadsheets for data collection

Figure 1D: General Information

Title	First Author	Publication year	Language	Developing / publishing organisation and / or authors	Country / region of publication	How described by the authors (e.g. guideline / consensus statement / position stateme	Version	Topic addressed (pain) [insert 1]	Topic addressed (sedation) [insert 1]	Topic addressed (delirium) [insert 1]	Topic addressed (withdrawal) [insert 1]	Target population (A+adult, C=child, NC=non-communicative)[insert all that apply]	Target setting (G=general; S=specific) [specify e.g. G (acute care) S (PICU)	LoE rating system	GoE rating system

Figure 2D: AGREE II

	CPG#1 [Name]		CPG#2 [Name]		CPG
**Domain 1: Scope and purpose**	Reviewer 1	Reviewer2	Reviewer 1	Reviewer2	Review
1. The overall objective(s) of the guideline is (are) specifically described					
2. The health question(s) covered by the guideline is (are) specifically described					
3. The population (patients, public, etc.) to whom the guideline is meant to apply is specifically described					
**Domain 2: Stakeholder involvement**					
4. The guideline development group includes individuals from all relevant professional groups					
5. The views and preferences of the target population (patients, public, etc.) have been sought					
6. The target users of the guideline are clearly defined					
**Domain 3: Rigour of development**					
7. Systematic methods were used to search for evidence					
8. The criteria for selecting the evidence are clearly described					
9. The strengths and limitations of the body of evidence are clearly described					
10. The methods for formulating the recommendations are clearly described					
11. The health benefits, side effects, and risks have been considered in formulating the recommendations					
12. There is an explicit link between the recommendations and the supporting evidence					
13. The guideline has been externally reviewed by experts prior to its publication					
14. A procedure for updating the guideline is provided					
**Domain 4: Clarity of presentation**					
15. The recommendations are specific and unambiguous					
16. The different options for management of the condition or health issue are clearly presented					
17. Key recommendations are easily identifiable					
**Domain 5: Applicability**					
18. The guideline describes facilitators and barriers to its application					
19. The guideline provides advice and/or tools on how the recommendations can be put into practice					
20. The potential resource implications of applying the recommendations have been considered					
21. The guideline presents monitoring and/or auditing criteria					
**Domain 6: Editorial independence**					
22. The views of the funding body have not influenced the content of the guideline					
23. Competing interests of guideline development group members have been recorded and addressed					
**Overall guideline assessment**					
1. Rate the overall quality of this guideline					

Figure 3D: AGREE-REX

Guideline Name	CPG#1 [Name]		CPG#2 [Name]		CPG#3 [Na	
**Domain 1: Clinical Applicability**	Reviewer 1	Reviewer2	Reviewer 1	Reviewer2	Reviewer 1	Rev
1. Evidence						
2. Applicability to Target Users						
3. Applicability to Patients/Populations						
**Domain 2: Values and Preferences**						
4. Values and Preferences of Target Users						
5. Values and Preferences of Patients/Populations						
6. Values and Preferences of Policy/Decision-Makers						
7. Values and Preferences of Guideline Developers						
**Domain 3: Implementability**						
8. Purpose						
9. Local Application and Adoption						
1. I would recommend these guideline recommendations for use in the appropriate context.						
2. I would recommend these guideline recommendations for use in my context (optional).						

review O

Figure 4D: Recommendations and evidence (one table per concept)

Guideline Title	Recommendation	Grade of Recommendation	Evidence cited for recommendation (Author, year)	Recommendation category specified within guideline

BMJ Paediatrics Open

Quality of clinical practice guidelines and recommendations for the management of pain, sedation, delirium, and iatrogenic withdrawal in pediatric intensive care: A systematic review protocol

Journal:	*BMJ Paediatrics Open*
Manuscript ID	bmjpo-2021-001293.R2
Article Type:	Protocol
Date Submitted by the Author:	10-Jan-2022
Complete List of Authors:	MacDonald, Ibo; University of Lausanne, University Institute of Higher Education and Research in Healthcare<br>Perez, Marie-Hélène; Lausanne University Hospital Department of Woman Mother and Child<br>Amiet, Vivianne; Lausanne University Hospital Department of Woman Mother and Child<br>Trombert, Alexia; Lausanne University Hospital, Medical Library<br>Ramelet, Anne-Sylvie; University of Lausanne Faculty of Biology and Medicine, University Institute of Higher Education and Research in Healthcare
Keywords:	Pain

SCHOLARONE™
Manuscripts

I, the Submitting Author has the right to grant and does grant on behalf of all authors of the Work (as defined in the below author licence), an exclusive licence and/or a non-exclusive licence for contributions from authors who are: i) UK Crown employees; ii) where BMJ has agreed a CC-BY licence shall apply, and/or iii) in accordance with the terms applicable for US Federal Government officers or employees acting as part of their official duties; on a worldwide, perpetual, irrevocable, royalty-free basis to BMJ Publishing Group Ltd ("BMJ") its licensees and where the relevant Journal is co-owned by BMJ to the co-owners of the Journal, to publish the Work in this journal and any other BMJ products and to exploit all rights, as set out in our licence.

The Submitting Author accepts and understands that any supply made under these terms is made by BMJ to the Submitting Author unless you are acting as an employee on behalf of your employer or a postgraduate student of an affiliated institution which is paying any applicable article publishing charge ("APC") for Open Access articles. Where the Submitting Author wishes to make the Work available on an Open Access basis (and intends to pay the relevant APC), the terms of reuse of such Open Access shall be governed by a Creative Commons licence – details of these licences and which Creative Commons licence will apply to this Work are set out in our licence referred to above.

Other than as permitted in any relevant BMJ Author's Self Archiving Policies, I confirm this Work has not been accepted for publication elsewhere, is not being considered for publication elsewhere and does not duplicate material already published. I confirm all authors consent to publication of this Work and authorise the granting of this licence.

Confidential: For Review Only

Quality of clinical practice guidelines and recommendations for the management of pain, sedation, delirium, and iatrogenic withdrawal in pediatric intensive care: A systematic review protocol

Ibo MacDonald[1], Marie-Hélène Perez[2], Vivianne Amiet[2], Alexia Trombert[3], Anne-Sylvie Ramelet[1,2]*

Full name	Institution, Department	Postal address		e-mail	ORCID ID
*Corresponding author					
Anne-Sylvie Ramelet	University Institute of Higher Education and Research in Healthcare, Faculty of Biology and Medicine, University of Lausanne and Department Woman-Mother-Child, Lausanne University Hospital	Route de la Corniche 10, Lausanne, Switzerland, CH-1010		Anne-sylvie.ramelet@chuv.ch	0000-0001-8809-2920
Co-authors	Institution, Department	City	Country	e-mail	
Ibo MacDonald	University Institute of Higher Education and Research in Healthcare, Faculty of Biology and Medicine, University of Lausanne	Lausanne	Switzerland	Ibo.macdonald@chuv.ch	0000-0001-9490-8850
Marie-Hélène Perez	Lausanne University Hospital, Department Woman-Mother-Child and University of Lausanne			Marie-helene.perez@chuv.ch	0000-0001-6173-3396
Vivianne Amiet	Lausanne University Hospital, Department Woman-Mother-Child and University of Lausanne			Vivianne.Amiet@chuv.ch	0000-0002-6665-5291
Alexia Trombert	Lausanne University Hospital and University of Lausanne, Medical Library			Alexia.Trombert@chuv.ch	0000-0002-4833-5219

**SUMMARY**<br>**Word count:** 2750/2500<br>**Abstract:** 295/300<br>**Tables/figures**: 1/8<br>**References:** 44/40	

Confidential: For Review Only

ABSTRACT (295/300)

Introduction: Inadequate management of pain and sedation in critically ill children can cause unnecessary suffering and agitation, but also delirium and iatrogenic withdrawal. It is therefore important to address these four interrelated conditions together. Some clinical practice guidelines (CPGs) are available for the management of pain and sedation, and a few for delirium and iatrogenic withdrawal in the PICU; none address the four conditions altogether. Critical appraisal of the quality of CPGs is necessary for their recommendations to be adopted into clinical practice. The aim of this systematic review is to identify and appraise the quality of CPGs and recommendations for management of either pain, sedation, delirium and iatrogenic withdrawal.

Methods and analysis: Researchers will conduct a systematic review in electronic databases (Medline ALL (Ovid), Embase.com, CINAHL with Full Text (EBSCO), JBI EBP Database (Ovid)), guideline repositories and websites of professional societies to identify CPGs published from 2010 to date. They will then combine index and free terms describing CPGs with pain, sedation, delirium and withdrawal. The researchers will include CPGs if they can be applied in the pediatric intensive care population (newborns to 18 years old) and include recommendation(s) for assessment of at least one of the four conditions. Two independent reviewers will screen for eligibility, complete data extraction and quality assessments using the Appraisal of Guidelines for Research and Evaluation (AGREE) II and the AGREE Recommendation Excellence (AGREE-REX) instruments. Researchers will report characteristics, content and recommendations from CPGs in tabulated forms.

Ethics and dissemination: Ethical approval is not required for this systematic review. Results will be published in a peer-reviewed journal.

Trial registration: This protocol is registered in the international database of prospectively registered systematic reviews (PROSPERO CRD42021274364).

Key words: AGREE II, children, clinical practice guideline, delirium, pain, sedation, systematic review, iatrogenic withdrawal

Confidential: For Review Only

SUMMARIZING BOX

**What is known about the subject** (2/3 statements)<br>• Optimal analgesia and sedation management is challenging in the pediatric intensive care unit. (13/25 words)<br>• Pain, sedation, delirium and iatrogenic withdrawal are interrelated conditions that need to be assessed as a whole to prevent negative outcomes. (21/25 words)
**What this study hopes to add** (2/3 statements)<br>• Researchers will compare the quality of clinical practice guidelines across all four interrelated conditions of pain, sedation, delirium and iatrogenic withdrawal. (21/25 words)<br>• Researchers will compare recommendations by describing the certainty and applicability of the base of evidence for clinical practice guidelines. (19/25 words)

Confidential: For Review Only

INTRODUCTION

Critically ill children in the pediatric intensive care unit (PICU), particularly those who are mechanically ventilated, require adequate identification and treatment of pain, sedation, delirium and iatrogenic withdrawal.[1] Healthcare professionals (HCPs) manage this with the use of analgesics and sedatives, often in combination, with 65% of children receiving both.[2] Optimal titration of analgesics and sedatives prevent pain, delirium and iatrogenic withdrawal,[3,4] as well as agitation that can cause accidental extubation.[5] Over sedation and analgesia can result in prolonged mechanical ventilation, increased PICU length of stay, morbidity and mortality.[1] In the PICU setting 40-65% of children are unable to self-report due to mechanical ventilation and young age (under the age of four).[6,7] Given the large proportion of non-communicative children and the need for quality care, it is crucial that HCPs use multiple measurement instruments to assess pain, sedation, delirium and iatrogenic withdrawal.

Assessment is the cornerstone for management of the four conditions.[8] It helps HCPs to individualize treatment and plan appropriate multi-modal interventions.[1] Although, HCPs have access to several measurement instruments for assessing pain and sedation,[9] and more recently, for delirium and iatrogenic withdrawal.[10] A recent survey including 168 PICUs in 18 countries, found wide variation in the application of measurement instruments across the four conditions into practice.[11] In fact, some researchers have shown that HCPs struggle to select the right measurement instrument for these four conditions.[12,13] This may be due to the overlap among similar behavioural cue items across measurement instruments across these four conditions, or the plethora of measurement instruments available.[10,14,15] While on one hand there is a need for psychometrically sound measurement instruments for each condition, it is equally important not to look at these conditions in siloes but to incorporate them together into standardized care practices,[14,15] either in clinical practice guidelines (CPGs) or as protocols or algorithms to improve assessment and management.

CPGs are created by synthesizing research to help bridge the evidence-to-practice gap. The purported benefits of CPG implementation are the standardization of care practices; improvements in patient safety; and patient outcomes.[16] There are few CPGs for management of these four conditions in the PICU, with only two for pain,[17,18] and one for pain, sedation and delirium.[19] Furthermore, some guidance documents exist, including practice/position/consensus statements/recommendations (hereafter, referred to as CPGs).[14,20,21] Although there is one position statement for the assessment of pain, sedation,

Confidential: For Review Only

delirium and iatrogenic withdrawal for pediatric critical care patients,[14] it does not include recommendations for management. Previous systematic reviews of CPGs for pain in pediatrics exist. They target neonates,[22] or burn patients,[23] or focus on procedural[22,24] or acute pain.[23] Systematic reviews of CPGs concerning best practices for children in the PICU are lacking. To date, none have been conducted on either of the four conditions, nor have they been examined together. Recently, scholars have criticized systematic reviews of CPGs and their lack of quality appraisal of recommendations.[25] Several systematic reviews of CPG recommendations have demonstrated evidence that weakly supports the recommendations.[26,27] Researchers, in a recent systematic review of CPGs for pediatric populations, that filtered publications between 2017 and 2019, found that 75% of the 216 CPGs were evidence-based.[28] It is important that researchers establish methodological quality of CPGs and the evidence base of recommendations to promote evidence-informed interventions.

Quality CPGs and their contained recommendations should reflect the most current evidence. Although, numerous appraisal instruments exist for assessing the quality of CPGs,[29] internationally, the appraisal of guidelines for research and evaluation (AGREE) II has emerged as the most widely used appraisal instrument.[30] The AGREE Enterprise recently developed the AGREE recommendation of excellence (AGREE-REX) instrument for the quality appraisal of recommendations.[31] Thus, researchers should use these two instruments jointly to assess CPG quality and to ensure that the evidence supporting recommendations in CPGs is reliable and trustworthy. This is essential so that CPGs remain sources of information that clinicians use to improve their practice and care of patients. The identification, appraisal and comparison of quality of CPGs and their recommendations is a valuable first step in informing efforts to incorporate these four overlapping conditions together in a standardized way to optimize care in the PICU. In this systematic review, researchers aim to appraise the quality of CPGs and recommendations for the assessment and management of pain, sedation, delirium and iatrogenic withdrawal in the PICU. Their specific objectives are:

1. To identify published CPGs for the assessment and management of pain, sedation, delirium and iatrogenic withdrawal;
2. To appraise the quality of selected CPGs;
3. To appraise the quality of recommendations included in CPGs; and
4. To summarize the convergence of recommendations and the overall robustness of recommendation in CPGs.

Confidential: For Review Only

METHODS AND ANALYSIS

Researchers of this study protocol used the methodological guide for conducting systematic reviews of CPGs to guide the development of each stage.[32] They reported according to the preferred reporting items for systematic review and meta-analysis protocols (PRISMA-P) (online supplemental table 1A).[33] The researchers registered the study protocol in the international database of prospectively registered systematic reviews (PROSPERO ID CRD42021274364, available from: https://www.crd.york.ac.uk/prospero/display_record.php?ID=CRD42021274364). In the event of amendment to this protocol, the PROSPERO register will be updated.

Inclusion/exclusion criteria for study selection

To guide CPG selection, the population, intervention, comparators, attributes and recommendations (PICAR) framework was used (see Table 1).[32] For the purpose of this review, CPGs must have included recommendations developed from available evidence, including expert opinion.[16] CPGs and guidance statements will be considered. CPGs that include pediatric populations will be included in this review if they: i) are endorsed by a society, ii) include a recommendation for assessment of any of the four conditions, and iii) are the most current version. Publication year will be limited to 2010 to present for two reasons. Firstly, this timeframe corresponds with a paradigmatic shift in intensive care unit sedation practice.[34] Secondly, the first consensus guideline for critically ill children was published in 2006.[20] If updated within 5 years, as recommended,[35] it would be captured within the search strategy. This review will include broad CPGs for the assessment and management of any of the four conditions, including postoperative pain. However, CPGs will be excluded if focused on specific patient groups (e.g. cardiac). CPGs on diagnostic procedures (e.g. endoscopy) or procedures of limited temporal duration (e.g. venipuncture) will be excluded. Procedures that require prolonged use in the PICU setting (e.g. respiratory support), will be included.

Table 1: PICAR statement: inclusion and exclusion criteria

Population, clinical indication(s), and condition(s)	**Study population**:<br>- Include: Children (newborn (>38 weeks gestations) to 18 years of age)<br>- Exclude: premature infants and adults<br>**Clinical indications**:<br>- Include: management of either pain (including postoperative, persistent and prolonged pain), sedation, delirium or iatrogenic withdrawal

Confidential: For Review Only

	- Exclude: Management specific to medications, chronic and procedural pain of short duration, procedural sedation provided in other care settings (e.g. dentistry, radiology, endoscopy) or for short duration<br>**Conditions**:<br>- Include: children in intensive care
**I**nterventions	Any intervention focusing on the on-going management of either pain, sedation, delirium or iatrogenic withdrawal
**C**omparator(s), comparison(s), and (key) content	**Comparator/comparison:** Any<br>**Key content**:<br>- If a broad population is included, the CPG must have separate recommendations for children<br>- The CPG can be implemented in the intensive care setting but does not need to be specifically developed for intensive care<br>- The CPG must include recommendations on assessment for either pain, sedation, delirium or iatrogenic withdrawal
**A**ttributes of eligible CPGs	**Language:** No restrictions<br>**Year of publication**: 2010 onward<br>**Setting**:<br>- Include: Applicable to pediatric intensive care, can be broad/general<br>- Exclude: CPGs developed specifically for other settings: neonatal intensive care units, emergency department, pre-operative/operating room<br>**Developing/publishing organization:**<br>- Include: CPGs issued or endorsed by international, national or regional societies/professional organizations, or governments from developed countries<br>- Exclude: CPGs that were developed by an individual organization (e.g. hospital) or unit within an organization<br>**Version**: Latest/newest version (preceding versions will be excluded)<br>**Type**: CPGs, consensus statements, practice/position recommendations/alerts/statements<br>**Quality score**: The AGREE II will be used to assess quality but will not be used as a criterion to determine eligibility for inclusion in this systematic review
**R**ecommendations characteristics and "other" considerations	**Recommendations:** CPGs must have at least one specific recommendation for assessment for either pain, sedation, delirium or iatrogenic withdrawal (either explicitly highlighted as a recommendation (primary) or noted in the body of the text (secondary - not explicitly identified as a recommendation))

126

127 ***Search methods***

128 *Informational sources*

129 The search will be conducted in:

Confidential: For Review Only

1) Four electronic databases: Medline ALL (Ovid), Embase.com CINAHL with Full Text (EBSCO), and Joanna Briggs Institute (JBI) EBP Database.
2) Ten guideline repositories.
3) Thirteen professional societies/organizations (online supplemental table 1B contains a list of guideline repositories and professional societies/organizations to be searched).

Search strategy

The search strategy will be developed with the assistance of a health services librarian. Index and free terms describing CPGs and pain, sedation, delirium, withdrawal will be combined to create an advanced search strategy that will be translated for all databases and sources of information. The final search strategy for Embase.com is provided in the online supplemental table 1C. The search strategy will be peer reviewed by another librarian using the PRESS checklist.[36] During full-text screening, if a CPG is mentioned, it will be retrieved for review.

Guideline selection

The search results will be imported into Endnote 20 reference manager (Clarivate Analytics, USA) for duplicate removal. The remaining citations will be uploaded to Rayyan QCRI (Qatar Computing Research Institute, Doha, Qatar) to manage the screening process.[37]

Titles and abstracts of all citations will be screened by two independent reviewers to determine those for full-text review. These will be retrieved and assessed against the inclusion and exclusion criteria. Reasons for exclusion will be recorded. Any disagreements will be resolved through discussion and consensus or by a third reviewer.

Supporting documents (e.g. evidence tables, conflict of interest declarations), where available, will be retrieved by the review team from the endorsing organization's website to ensure all relevant documents will be available for quality appraisal for included CPGs.

The PRISMA flow diagram will be used to show the selection process and summarise the inclusion and exclusion details.[38]

Data extraction

Information from each included CPGs will be extracted by two independent reviewers. The review team developed an Excel spreadsheet for data extraction (online supplemental figures 1D

Confidential: For Review Only

159 to 4D) that will be piloted and revised during the data extraction phase. The following key areas will be extracted:

a) General information: title, first author, year of publication, language, developing organization, country, type of CPG, condition addressed (pain, sedation, delirium, iatrogenic withdrawal), target population, target setting, level of evidence (LoE) rating system, grade of evidence (GoE) rating system.
b) Quality of included CPGs using the AGREE II instrument (details below).[30]
c) Quality of recommendations from medium quality and higher CPGs using the AGREE-REX instrument (details below).[31]
d) Recommendations (one worksheet per condition, and each line will represent one recommendation): recommendation, grade of recommendation, classification of evidence, list of supporting citation(s), categorization of recommendation as per CPG.

Quality appraisal of CPGs and recommendations

Quality of CPGs. Each included CPG will be independently appraised by at least two reviewers using the AGREE II instrument.[30] The AGREE II is a validated and reliable appraisal instrument for assessing the quality of CPGs.[30] It contains 23 items across six domains: 1) scope and purpose; 2) stakeholder involvement; 3) rigour of development; 4) clarity of presentation; 5) applicability; and 6) editorial independence. Each item will be appraised against a 7-point Likert scale ranging from 1 (strongly disagree) to 7 (strongly agree). In addition, there are two global rating scores: 1) overall quality of the CPG, and 2) whether the guideline would be recommended for use.

Quality of CPGs recommendations. The AGREE–REX will be used for assessing the quality of recommendations.[31] It is a recently developed, valid and reliable appraisal instrument containing nine items across three domains: 1) clinical applicability; 2) values and preferences; and 3) implementability.[31] Each item will be appraised by at least three reviewers using the same seven-point Likert scale, as in the AGREE II. It includes a global rating score of the overall quality of the CPGs recommendations.

To score the AGREE II each item’s score across reviewers will be summed and converted to a percentage of the maximum possible score for each domain.[30] For the AGREE-REX, the consensus score approach will be used, whereby the review team will meet to agree on AGREE-REX item scores [39]

To ensure standardization of appraisal, the training tools available for the AGREE II on the website (www.agreetrust.com) will be used to train each reviewer. For the AGREE-REX, a training video will be created by one research team member (IMD). This video will be used to train each member of the review team. One included CPG will then be selected by the entire review team, and a consensus meeting will be held to ensure familiarity with the tools. The AGREE II inter-rater agreement will be calculated using intra-class correlation coefficients (ICCs) with a two-way random effects model for each domain. The levels of ICC agreement will be classified as poor (<0.50), moderate (0.50–0.75), good (0.75–0.9), and excellent (>0.9).[40]

Data synthesis

Quality of CPGs and recommendations

The AGREE Enterprise has no established quality threshold, instead review teams must establish their own prior to appraisal.[41] As recommended in a recent systematic review of AGREE II thresholds for determining CPG quality, we will use the 3-step system where high quality are scores ≥ 60%, medium quality are scores between ≥30% - 60%, and low quality are scores < 30% across all domains.[42] For determining when the AGREE-REX will be applied to assessing recommendations the same a priori establishment of a threshold is recommended in the AGREE-REX user manual [41]. For this review, the AGREE-REX will be used only with CPGs that meet at least the medium level threshold (e.g. ≥ 30%) for methodological development using the AGREE II. Clusters of recommendations on single topics (e.g. assessment) will then be appraised in these CPGs. This decision was made because this is the first review of its kind, and the quality of recommendations between CPGs is uncertain.

The results of the AGREE II and AGREE-REX scores will be presented in a table. The quality of each domain will be presented as a heat map based on the threshold cut-offs for quality as described above.

Synthesis of recommendations and their level of evidence

For medium and high methodological quality CPGs (based on the AGREE II), all recommendations and their related evidence will be extracted per condition. Once extracted, each recommendation will be categorized based on the type of care intervention, including: a) prevention, b) assessment, and c) management. Management interventions will be further subdivided into pharmacological and non-pharmacological. Summary tables will be created to

Confidential: For Review Only

highlight the consistency of all recommendations for each condition. The LoE associated with recommendations within each CPG will be reported but not standardized across CPGs.

ETHICS AND DISSEMINATION

Ethical approval is not required for this systematic review. To disseminate the results the study will be published in a peer-reviewed journal.

PATIENT AND PUBLIC INVOLVEMENT

There was no patient or public involvement in the development of the systematic review protocol. The Swiss Society of Intensive Care Medicines' Pain, Agitation, Delirium, Immobility and Sleep working group (Swiss PADIS-WG) will be involved in data synthesis as clinical experts.

DISCUSSION

This systematic review of CPGs will generate a succinct and comprehensive summary of the best available evidence for the assessment and management of pain, sedation, delirium and iatrogenic withdrawal. This will be a valuable first step towards standardizing the assessment and management of pain, sedation, delirium and iatrogenic withdrawal in the PICU.

Research on pain, sedation, delirium and iatrogenic withdrawal practices across an international sample of 161 PICUs continues to demonstrate great variation.[11] Although HCPs use measurement instruments to identify patient changes based on behavioural cues, HCPs may find it challenging to interpret and use scores and determine which multi-modal interventions to use. The overlap among similar behavioural cue items across measurement instruments and the multiple measurement instruments available has proved challenging for HCPs.[13,14] Pain and sedation, and delirium and withdrawal, are concomitant pairs. This is demonstrated by the development and use of measurement instruments for these pairs (e.g. COMFORT behaviour scale[43] and SOS-PD[44]). Using measurement instruments is the first step towards goal-directed care, and this review will synthesize strategies to inform clinical practice.

Based on quality appraisal, the results will establish which CPGs can be recommended for use and implementation into clinical practice. It will also provide accessible summaries of the best evidence for each recommendation and type of care intervention for the four conditions to

Confidential: For Review Only

support implementation into practice. These results can be used as the basis for the development of a combined CPG for these four conditions specific for the PICU.

The strengths of this systematic review are the comprehensive search for CPGs on the four interrelated conditions, which has not been previously conducted, as well as the evaluation of the quality of CPGs and the base of evidence for included recommendations.

A limitation of this systematic review will be ensuring the review team is sufficiently trained in using the AGREE-REX, as this is a novel tool. Currently, no training resources exist and guidance on its use during the systematic review process is lacking. This might lead to divergent scores. To mitigate this limitation, a member of the review team will develop a training video, and the researchers will use a consensus process. It is likely that the heterogeneity of research conducted in the PICU setting will lead to low-quality scores for recommendations.

CONCLUSION

Management of pain and sedation is a balancing act for HCPs in order to provide optimal comfort and avoid delirium and iatrogenic withdrawal for their pediatric patients. Recommendations for managing these four interrelated conditions are mixed. This systematic review will use rigorous methods to assess the quality and content of CPGs and included recommendations for the assessment and management of these four conditions. It will add to the current body of knowledge with the intention to optimise care and outcomes for critically ill pediatric patients in the PICU.

AUTHOR CONTRIBUTIONS.

The conception and design of this systematic review protocol was completed by three authors (IMD, ASR and MHP). The search strategy was developed by IMD and AT with feedback from the review team (ASR, MHP and VA). IMD prepared the first draft of the manuscript with all authors providing feedback towards the final manuscript.

FUNDING STATEMENT.

This research received no specific grant from any funding agency in the public, commercial or not-for-profit sectors.

COMPETING INTERESTS.

None declared

Confidential: For Review Only

REFERENCES

1. Egbuta C, Mason KP. Current State of Analgesia and Sedation in the Pediatric Intensive Care Unit. *J Clin Med* 2021;10(9):1847. doi: 10.3390/jcm10091847 [published Online First: 2021/05/01]
2. Patel AK, Trujillo-Rivera E, Faruqe F, et al. Sedation, Analgesia, and Neuromuscular Blockade: An Assessment of Practices From 2009 to 2016 in a National Sample of 66,443 Pediatric Patients Cared for in the ICU. *Pediatr Crit Care Med* 2020;21(9):e599-e609. doi: 10.1097/PCC.0000000000002351 [published Online First: 2020/03/21]
3. Best KM, Boullata JI, Curley MA. Risk factors associated with iatrogenic opioid and benzodiazepine withdrawal in critically ill pediatric patients: a systematic review and conceptual model. *Pediatr Crit Care Med* 2015;16(2):175-83. doi: 10.1097/PCC.0000000000000306 [published Online First: 2015/01/07]
4. Mody K, Kaur S, Mauer EA, et al. Benzodiazepines and Development of Delirium in Critically Ill Children: Estimating the Causal Effect. *Crit Care Med* 2018;46(9):1486-91. doi: 10.1097/CCM.0000000000003194 [published Online First: 2018/05/05]
5. Lucas da Silva PS, de Carvalho WB. Unplanned extubation in pediatric critically ill patients: a systematic review and best practice recommendations. *Pediatr Crit Care Med* 2010;11(2):287-94. doi: 10.1097/PCC.0b013e3181b80951 [published Online First: 2009/10/02]
6. Ibiebele I, Algert CS, Bowen JR, et al. Pediatric admissions that include intensive care: a population-based study. *BMC Health Serv Res* 2018;18(1):264. doi: 10.1186/s12913-018-3041-x [published Online First: 2018/04/11]
7. Laures E, LaFond C, Hanrahan K, et al. Pain Assessment Practices in the Pediatric Intensive Care Unit. *J Pediatr Nurs* 2019;48:55-62. doi: 10.1016/j.pedn.2019.07.005 [published Online First: 2019/07/22]
8. Stonehouse D. Understanding the nursing process. *British Journal of Healthcare Assistants* 2017;11(8):388-91. doi: 10.12968/bjha.2017.11.8.388Giordano V, Edobor J, Deindl P, et al. Pain and Sedation Scales for Neonatal and Pediatric Patients in a Preverbal Stage of Development: A Systematic Review. *JAMA Pediatr* 2019;173(12):1186-97. doi: 10.1001/jamapediatrics.2019.3351 [published Online First: 2019/10/15]
9. Giordano V, Edobor J, Deindl P, et al. Pain and Sedation Scales for Neonatal and Pediatric Patients in a Preverbal Stage of Development: A Systematic Review. *JAMA Pediatr* 2019;173(12):1186-97. doi: 10.1001/jamapediatrics.2019.3351 [published Online First: 2019/10/15]
10. Madden K, Burns MM, Tasker RC. Differentiating Delirium From Sedative/Hypnotic-Related Iatrogenic Withdrawal Syndrome: Lack of Specificity in Pediatric Critical Care Assessment Tools. *Pediatr Crit Care Med* 2017;18(6):580-88. doi: 10.1097/PCC.0000000000001153 [published Online First: 2017/04/22]
11. Ista E, Redivo J, Kananur P, et al. Assessing Pain, Both Spontaneous Awakening and Breathing Trials, Choice of Sedation, Delirium Monitoring/Management, Early Exercise/Mobility, and Family Engagement/Empowerment Bundle Practices for Critically Ill Children: An International Survey of 161 PICUs in 18 Countries. *Critical Care Medicine* 2021 doi: 10.1097/CCM.0000000000005168 [published Online First: July 13, 2021]
12. Flaigle MC, Ascenzi J, Kudchadkar SR. Identifying Barriers to Delirium Screening and Prevention in the Pediatric ICU: Evaluation of PICU Staff Knowledge. *J Pediatr Nurs* 2016;31(1):81-84. doi: https://doi.org/10.1016/j.pedn.2015.07.009
13. LaFond CM, Hanrahan KS, Pierce NL, et al. Pain in the pediatric intensive care unit: How and what are we doing? *American Journal of Critical Care* 2019;28(4):265-73. doi: 10.4037/ajcc2019836
14. Harris J, Ramelet AS, van Dijk M, et al. Clinical recommendations for pain, sedation, withdrawal and delirium assessment in critically ill infants and children: an ESPNIC position statement for healthcare

Confidential: For Review Only

professionals. *Intensive Care Med* 2016;42(6):972-86. doi: 10.1007/s00134-016-4344-1 [published Online First: 2016/04/17]

15. Ista E, van Dijk M. We can not compartmentalize our patients! Overlapping symptoms of iatrogenic withdrawal syndrome, pediatric delirium, and anticholinergic toxidrome. *Pediatric critical care medicine* 2017;18(6):603-04.doi: 10.1097/PCC.0000000000001163
16. Graham R. Clinical practice guidelines we can trust. Washington, DC: National Academies Press 2011.
17. Association of Paediatric Anaesthetists of Great B, Ireland. Good practice in postoperative and procedural pain management, 2nd edition. *Paediatr Anaesth* 2012;22 Suppl 1(s1):1-79. doi: 10.1111/j.1460-9592.2012.03838.x [published Online First: 2012/08/01]
18. Royal College of Nursing, editor. *The recognition and assessment of acute pain in children: update of full guideline [Electronic version]*, 2009.
19. Taskforce DAS, Baron R, Binder A, et al. Evidence and consensus based guideline for the management of delirium, analgesia, and sedation in intensive care medicine. Revision 2015 (DAS-Guideline 2015) - short version. *Ger Med Sci* 2015;13:Doc19. doi: 10.3205/000223 [published Online First: 2015/11/27]
20. Playfor S, Jenkins I, Boyles C, et al. Consensus guidelines on sedation and analgesia in critically ill children. *Intensive Care Med* 2006;32(8):1125-36. doi: 10.1007/s00134-006-0190-x [published Online First: 2006/05/16]
21. Sauder P, Andreoletti M, Cambonie G, et al. [Sedation and analgesia in intensive care (with the exception of new-born babies). French Society of Anesthesia and Resuscitation. French-speaking Resuscitation Society]. *Ann Fr Anesth Reanim* 2008;27(7-8):541-51. doi: 10.1016/j.annfar.2008.04.021 [published Online First: 2008/06/27]
22. Balice-Bourgois C, Zumstein-Shaha M, Vanoni F, et al. A Systematic Review of Clinical Practice Guidelines for Acute Procedural Pain on Neonates. *Clin J Pain* 2020;36(5):390-98. doi: 10.1097/AJP.0000000000000808 [published Online First: 2020/01/25]
23. Gamst-Jensen H, Vedel PN, Lindberg-Larsen VO, et al. Acute pain management in burn patients: appraisal and thematic analysis of four clinical guidelines. *Burns* 2014;40(8):1463-9. doi: 10.1016/j.burns.2014.08.020 [published Online First: 2014/10/04]
24. Lee GY, Yamada J, Kyololo O, et al. Pediatric clinical practice guidelines for acute procedural pain: a systematic review. *Pediatrics* 2014;133(3):500-15. doi: 10.1542/peds.2013-2744 [published Online First: 2014/02/04]
25. Eikermann M, Holzmann N, Siering U, et al. Tools for assessing the content of guidelines are needed to enable their effective use--a systematic comparison. *BMC Res Notes* 2014;7(1):853. doi: 10.1186/1756-0500-7-853 [published Online First: 2014/11/28]
26. McCurtin A, Boland P, Kavanagh M, et al. Do stroke clinical practice guideline recommendations for the intervention of thickened liquids for aspiration support evidence based decision making? A systematic review and narrative synthesis. *J Eval Clin Pract* 2020;26(6):1744-60. doi: 10.1111/jep.13372 [published Online First: 2020/02/23]
27. Cosgrove L, Bursztajn HJ, Erlich DR, et al. Conflicts of interest and the quality of recommendations in clinical guidelines. *J Eval Clin Pract* 2013;19(4):674-81. doi: 10.1111/jep.12016 [published Online First: 2013/06/05]
28. Liu Y, Zhang Y, Wang S, et al. Quality of pediatric clinical practice guidelines. *BMC Pediatr* 2021;21(1):223. doi: 10.1186/s12887-021-02693-1 [published Online First: 2021/05/09]
29. Siering U, Eikermann M, Hausner E, et al. Appraisal tools for clinical practice guidelines: a systematic review. *PLoS One* 2013;8(12):e82915. doi: 10.1371/journal.pone.0082915 [published Online First: 2013/12/19]

Confidential: For Review Only

30. Brouwers MC, Kho ME, Browman GP, et al. AGREE II: advancing guideline development, reporting and evaluation in health care. *CMAJ* 2010;182(18):E839-42. doi: 10.1503/cmaj.090449 [published Online First: 2010/07/07]
31. Brouwers MC, Spithoff K, Kerkvliet K, et al. Development and Validation of a Tool to Assess the Quality of Clinical Practice Guideline Recommendations. *JAMA Netw Open* 2020;3(5):e205535. doi: 10.1001/jamanetworkopen.2020.5535 [published Online First: 2020/05/28]
32. Johnston A, Kelly SE, Hsieh SC, et al. Systematic reviews of clinical practice guidelines: a methodological guide. *J Clin Epidemiol* 2019;108:64-76. doi: 10.1016/j.jclinepi.2018.11.030 [published Online First: 2018/12/12]
33. Moher D, Shamseer L, Clarke M, et al. Preferred reporting items for systematic review and meta-analysis protocols (PRISMA-P) 2015 statement. *Syst Rev* 2015;4(1):1. doi: 10.1186/2046-4053-4-1 [published Online First: 2015/01/03]
34. Devabhakthuni S, Armahizer MJ, Dasta JF, et al. Analgosedation: a paradigm shift in intensive care unit sedation practice. *Ann Pharmacother* 2012;46(4):530-40. doi: 10.1345/aph.1Q525 [published Online First: 2012/04/13]
35. Vernooij RW, Sanabria AJ, Sola I, et al. Guidance for updating clinical practice guidelines: a systematic review of methodological handbooks. *Implement Sci* 2014;9(1):3. doi: 10.1186/1748-5908-9-3 [published Online First: 2014/01/05]
36. McGowan J, Sampson M, Salzwedel DM, et al. PRESS Peer Review of Electronic Search Strategies: 2015 Guideline Statement. *J Clin Epidemiol* 2016;75:40-6. doi: 10.1016/j.jclinepi.2016.01.021 [published Online First: 2016/03/24]
37. Ouzzani M, Hammady H, Fedorowicz Z, et al. Rayyan-a web and mobile app for systematic reviews. *Syst Rev* 2016;5(1):210. doi: 10.1186/s13643-016-0384-4 [published Online First: 2016/12/07]
38. Page MJ, Moher D, Bossuyt PM, et al. PRISMA 2020 explanation and elaboration: updated guidance and exemplars for reporting systematic reviews. *BMJ* 2021;372:n160. doi: 10.1136/bmj.n160 [published Online First: 2021/03/31]
39. AGREE-REX Research Team. The Appraisal of Guidelines Research & Evaluation – Recommendation Excellence (AGREE-REX) Instruction Manual. 2019.
40. Koo TK, Li MY. A Guideline of Selecting and Reporting Intraclass Correlation Coefficients for Reliability Research. *J Chiropr Med* 2016;15(2):155-63. doi: 10.1016/j.jcm.2016.02.012 [published Online First: 2016/06/23]
41. The AGREE Next Steps Consortium. Appraisal of guidelines for research and evaluation II instrument - user manual. Ontario, CA: The Agree Research Trust 2010.
42. Hoffmann-Esser W, Siering U, Neugebauer EAM, et al. Systematic review of current guideline appraisals performed with the Appraisal of Guidelines for Research & Evaluation II instrument-a third of AGREE II users apply a cut-off for guideline quality. *J Clin Epidemiol* 2018;95:120-27. doi: 10.1016/j.jclinepi.2017.12.009 [published Online First: 2017/12/31]
43. van Dijk M, de Boer JB, Koot HM, et al. The reliability and validity of the COMFORT scale as a postoperative pain instrument in 0 to 3-year-old infants. *Pain* 2000;84(2):367-77. doi: 10.1016/s0304-3959(99)00239-0
44. Ista E, Te Beest H, van Rosmalen J, et al. Sophia Observation withdrawal Symptoms-Paediatric Delirium scale: A tool for early screening of delirium in the PICU. *Australian critical care : official journal of the Confederation of Australian Critical Care Nurses* 2018;31(5):266-73. doi: 10.1016/j.aucc.2017.07.006 [published Online First: 2017/08/28]

Confidential: For Review Only

SUPPLEMENTARY MATERIALS

Table 1A: PRISMA-P[1] (Preferred Reporting Items for Systematic review and Meta-Analysis Protocols) 2015 checklist: recommended items to address in a systematic review protocol

Section and topic	Item No	Checklist item	Page number (line)
**ADMINISTRATIVE INFORMATION**			
Title:			
Identification	1a	Identify the report as a protocol of a systematic review	6 (105 - 112) + title
Update	1b	If the protocol is for an update of a previous systematic review, identify as such	N/A
Registration	2	If registered, provide the name of the registry (such as PROSPERO) and registration number	Abstract + 6 (108-112)
Authors:			
Contact	3a	Provide name, institutional affiliation, e-mail address of all protocol authors; provide physical mailing address of corresponding author	1 (3-5 +table)
Contributions	3b	Describe contributions of protocol authors and identify the guarantor of the review	12 (270 – 273)
Amendments	4	If the protocol represents an amendment of a previously completed or published protocol, identify as such and list changes; otherwise, state plan for documenting important protocol amendments	6 (111-112)
Support:			
Sources	5a	Indicate sources of financial or other support for the review	12 (275-276)
Sponsor	5b	Provide name for the review funder and/or sponsor	N/A
Role of sponsor or funder	5c	Describe roles of funder(s), sponsor(s), and/or institution(s), if any, in developing the protocol	N/A
**INTRODUCTION**			
Rationale	6	Describe the rationale for the review in the context of what is already known	4-5 (36 -95)
Objectives	7	Provide an explicit statement of the question(s) the review will address with reference to participants, interventions, comparators, and outcomes (PICO)	5-6 (95-103)
**METHODS**			
Eligibility criteria	8	Specify the study characteristics (such as PICO, study design, setting, time frame) and report characteristics (such as years considered, language, publication status) to be used as criteria for eligibility for the review	6-7 (113-128; Table 1: PICAR)
Information sources	9	Describe all intended information sources (such as electronic databases, contact with study authors, trial registers or other grey literature sources) with planned dates of coverage	8 (132 – 139) (supplement table 1B)

Confidential: For Review Only

Search strategy	10	Present draft of search strategy to be used for at least one electronic database, including planned limits, such that it could be repeated	8 (140-146) (supplement table 1C)
Study records:			
Data management	11a	Describe the mechanism(s) that will be used to manage records and data throughout the review	8 (148 – 150)
Selection process	11b	State the process that will be used for selecting studies (such as two independent reviewers) through each phase of the review (that is, screening, eligibility and inclusion in meta-analysis)	8 (150 – 157)
Data collection process	11c	Describe planned method of extracting data from reports (such as piloting forms, done independently, in duplicate), any processes for obtaining and confirming data from investigators	9 (161 – 164)
Data items	12	List and define all variables for which data will be sought (such as PICO items, funding sources), any pre-planned data assumptions and simplifications	9 (165 – 174) (supplement tables 1D – 4D)
Outcomes and prioritization	13	List and define all outcomes for which data will be sought, including prioritization of main and additional outcomes, with rationale	9 - 10 (190-193)
Risk of bias in individual studies	14	Describe anticipated methods for assessing risk of bias of individual studies, including whether this will be done at the outcome or study level, or both; state how this information will be used in data synthesis	9 (176-189)
Data synthesis	15a	Describe criteria under which study data will be quantitatively synthesized	N/A
	15b	If data are appropriate for quantitative synthesis, describe planned summary measures, methods of handling data and methods of combining data from studies, including any planned exploration of consistency (such as I^2, Kendall's τ)	N/A
	15c	Describe any proposed additional analyses (such as sensitivity or subgroup analyses, meta-regression)	N/A
	15d	If quantitative synthesis is not appropriate, describe the type of summary planned	10-11 (216 – 226)
Meta-bias(es)	16	Specify any planned assessment of meta-bias(es) (such as publication bias across studies, selective reporting within studies)	None
Confidence in cumulative evidence	17	Describe how the strength of the body of evidence will be assessed (such as GRADE)	11 (205 - 215)

N/A = not applicable

[1] Moher D, Shamseer L, Clarke M, et al. Preferred reporting items for systematic review and meta-analysis protocols (PRISMA-P) 2015 statement. *Systematic reviews* 2015;4(1):1.

Confidential: For Review Only

Table 1B: Guideline repositories and Professional Societies/Organizations

	Country/Region	Website
**Guideline repositories**		
Australian Clinical Practice Guidelines Portal	Australia	https://www.clinicalguidelines.gov.au/portal
BIGG International database of GRADE guidelines	Worldwide	https://sites.bvsalud.org/bigg/en/biblio/
CISMeF Bonnes pratiques	France	https://doccismef.chu-rouen.fr/dc/#env=bp
ECRI Guidelines Trust	USA/UK	https://guidelines.ecri.org/
Guideline Central	USA	https://www.guidelinecentral.com/summaries/#summary-view-specialty
Guidelines International Network (G-I-N)	Scotland	https://guidelines.ebmportal.com/
Infobanque Guides de Pratiques Clinique (GPC)	Canada	https://jouleamc.ca/cpg/homepage
NICE Evidence Search	UK	https://www.evidence.nhs.uk/
TRIP	UK	https://www.tripdatabase.com/
Up-to-date Society guideline links	Netherlands	http://www.uptodate.com/contents/search
**Professional societies/organizations**		
American Association of Critical-care Nurses	USA	https://www.aacn.org/clinical-resources/view-all-issues?category=practice-alerts
European Society of Intensive Care Medicine (ESICM)	Europe	https://www.esicm.org/

Confidential: For Review Only

European Society of Paediatric Neonatal Intensive Care (ESPNIC)	Europe	https://espnic-online.org
National Institute for Health and Care Excellence (NICE)	UK	www.nice.org.uk
Registered Nurses' Association of Ontario (RNAO)	Canada	https://rnao.ca/bpg
Royal College of Nursing	UK	https://www.rcn.org.uk/
Scottish Intercollegiate Guidelines Network (SIGN)	Scotland	http://sign.ac.uk/index.html
Société de Réanimation de Langue Française (SRLF)	France	https://srlf.org
Société Française d'Anesthésie et de Réanimation (SFAR)	France	https://sfar.org
Société Suisse de Pédiatrie	Switzerland	https://www.paediatrieschweiz.ch/fr/
Société Suisse de Médecine Intensive (SSMI)	Switzerland	https://www.sgi-ssmi.ch/fr
Society of Critical Care Medicine (SCCM)	USA	https://www.sccm.org/Home
World Federation of Pediatric Intensive and Critical Care Societies (WFPICCS)	Worldwide	httsp://wfpiccs.org

Confidential: For Review Only

Table 1C: Final search strategy for Embase.com

('pain'/de OR 'postoperative pain'/de OR 'analgesia'/exp OR 'conscious sedation'/de OR 'hypnotic sedative agent'/de OR 'delirium'/de OR 'hyperactive delirium'/de OR 'hypoactive delirium'/de OR 'withdrawal syndrome'/de OR (pain OR discomfort OR analgesia OR sedation OR sedative* OR delirium OR delirious* OR withdrawal*):ti,kw) AND ('practice guideline'/de OR 'consensus development'/de OR ((expert* NEAR/3 opinion*) OR guideline* OR cpg* OR guidance OR ((position OR policy) NEAR/3 (paper* OR development)) OR ((practice OR clinical) NEAR/3 development) OR (practice NEAR/2 guide$) OR recommendation* OR consensus OR standards OR statement*):ti,kw) NOT ('adult'/exp NOT 'juvenile'/exp) AND [2010-3000]/py NOT ('conference abstract'/it OR 'conference review'/it) NOT ([animals]/lim NOT [humans]/lim)
9849 references (17 November 2021)

Confidential: For Review Only

Figures 1D – 4D: Figures of excel spreadsheets for data collection

Figure 1D: General Information

Title	First Author	Publication year	Language	Developing / publishing organisation and / or authors	Country / region of publication	How described by the authors (e.g. guideline / consensus statement / position stateme	Version	Topic addressed (pain) [insert 1]	Topic addressed (sedation) [insert 1]	Topic addressed (delirium) [insert 1]	Topic addressed (withdrawal) [insert 1]	Target population (A+adult, C=child, NC=non-communicative)[insert all that apply)	Target setting (G=general; S=specific) [specify e.g. G (acute care) S (PICU)	LoE rating system	GoE rating system

Figure 2D: AGREE II

	CPG#1 [Name]		CPG#2 [Name]		CPG
**Domain 1: Scope and purpose**	Reviewer 1	Reviewer2	Reviewer 1	Reviewer2	Review
1. The overall objective(s) of the guideline is (are) specifically described					
2. The health question(s) covered by the guideline is (are) specifically described					
3. The population (patients, public, etc.) to whom the guideline is meant to apply is specifically described					
**Domain 2: Stakeholder involvement**					
4. The guideline development group includes individuals from all relevant professional groups					
5. The views and preferences of the target population (patients, public, etc.) have been sought					
6. The target users of the guideline are clearly defined					
**Domain 3: Rigour of development**					
7. Systematic methods were used to search for evidence					
8. The criteria for selecting the evidence are clearly described					
9. The strengths and limitations of the body of evidence are clearly described					
10. The methods for formulating the recommendations are clearly described					
11. The health benefits, side effects, and risks have been considered in formulating the recommendations					
12. There is an explicit link between the recommendations and the supporting evidence					
13. The guideline has been externally reviewed by experts prior to its publication					
14. A procedure for updating the guideline is provided					
**Domain 4: Clarity of presentation**					
15. The recommendations are specific and unambiguous					
16. The different options for management of the condition or health issue are clearly presented					
17. Key recommendations are easily identifiable					
**Domain 5: Applicability**					
18. The guideline describes facilitators and barriers to its application					
19. The guideline provides advice and/or tools on how the recommendations can be put into practice					
20. The potential resource implications of applying the recommendations have been considered					
21. The guideline presents monitoring and/or auditing criteria					
**Domain 6: Editorial independence**					
22. The views of the funding body have not influenced the content of the guideline					
23. Competing interests of guideline development group members have been recorded and addressed					
**Overall guideline assessment**					
1. Rate the overall quality of this guideline					

Figure 3D: AGREE-REX

Guideline Name	CPG#1 [Name]		CPG#2 [Name]		CPG#3 [Na	
**Domain 1: Clinical Applicability**	Reviewer 1	Reviewer2	Reviewer 1	Reviewer2	Reviewer 1	Rev
1. Evidence						
2. Applicability to Target Users						
3. Applicability to Patients/Populations						
**Domain 2: Values and Preferences**						
4. Values and Preferences of Target Users						
5. Values and Preferences of Patients/Populations						
6. Values and Preferences of Policy/Decision-Makers						
7. Values and Preferences of Guideline Developers						
**Domain 3: Implementability**						
8. Purpose						
9. Local Application and Adoption						
1. I would recommend these guideline recommendations for use in the appropriate context.						
2. I would recommend these guideline recommendations for use in my context (optional).						

Figure 4D: Recommendations and evidence (one table per concept)

Guideline Title	Recommendation	Grade of Recommendation	Evidence cited for recommendation (Author, year)	Recommendation category specified within guideline
